# Research progress of natural silk fibroin and the application for drug delivery in chemotherapies

**DOI:** 10.3389/fphar.2022.1071868

**Published:** 2023-01-04

**Authors:** Bin Yu, Yanli Li, Yuxian Lin, Yuanying Zhu, Teng Hao, Yan Wu, Zheng Sun, Xin Yang, Hui Xu

**Affiliations:** ^1^ School of Pharmacy, Collaborative Innovation Center of Advanced Drug Delivery System and Biotech Drugs in Universities of Shandong, Key Laboratory of Molecular Pharmacology and Drug Evaluation, Ministry of Education, Yantai University, Yantai, China; ^2^ Department of Pharmacy, Binzhou Hospital of Traditional Chinese Medicine, Binzhou, China; ^3^ Department of Pharmacy, Wenzhou People’s Hospital of The Third Affiliated Hospital of Shanghai University, The Third Clinical Institute Affiliated To Wenzhou Medical University, Wenzhou, China; ^4^ School of Chemistry and Chemical Engineering, Yantai University, Yantai, China

**Keywords:** silk fibroin, drug carrier, drug delivery system, cytotoxic agents, chemotherapy

## Abstract

Silk fibroin has been widely used in biological fields due to its biocompatibility, mechanical properties, biodegradability, and safety. Recently, silk fibroin as a drug carrier was developed rapidly and achieved remarkable progress in cancer treatment. The silk fibroin-based delivery system could effectively kill tumor cells without significant side effects and drug resistance. However, few studies have been reported on silk fibroin delivery systems for antitumor therapy. The advancement of silk fibroin-based drug delivery systems research and its applications in cancer therapy are highlighted in this study. The properties, applications, private opinions, and future prospects of silk fibroin carriers are discussed to understand better the development of anti-cancer drug delivery systems, which may also contribute to advancing silk fibroin innovation.

## 1 Introduction

Cancer remains the leading cause of death in humans. According to the “Global Cancer Statistics 2020’’ report, the average cancer morbidity and mortality have increased to 201.0/100,000 and 100.7/100,000 worldwide (Cao et al., 2021). Besides, according to estimates by the World Health Organization, the number of new cancer cases worldwide will exceed 27 million by 2040, with 1 in 5 people who have cancer, with an incidence rate of about 20% and a mortality rate of close to 11% (Sung et al., 2021). Hence, cancer seriously threaten human health. Various cancer treatment methods, including surgery, radiotherapy, chemotherapy, and immunotherapy, have been developed up to this point. However, limitations remain in overcoming tumor recurrence and metastasis. Moreover, there are other problems with these methods. For example, surgery is invasive; radiotherapy lacks specificity; chemotherapy fails to protect normal cells; and immunotherapy is less effective (Tulay et al., 2018; Nuvola et al., 2022). Therefore, the greatest challenge that has to be solved immediately now is to treat tumors effectively and safely, improve patient prognosis, and extend patient survival time.

Cytotoxic drugs are well-known anti-tumor drugs, such as cytarabine, gemcitabine, docetaxel, and paclitaxel. It has a very strong killing effect on tumor cells but will also kill normal cells in the human body (Al Alawi et al., 2020). This is especially obvious for the increase of toxic and side effects and the decline of quality of life in the later stage of patients. Therefore, reducing their inhibition and killing effects on normal cells and improving their targeting and therapeutic effects on tumor cells are the focus of current research. In recent years, research on drug preparation oriented to precise drug delivery is expected to solve the problem of cytotoxic drugs. It is of great value to develop new pharmaceutical strategies for tumor treatment, and the establishment and application of new drug delivery systems are exceptionally prominent for improving cytotoxic drug problems (Mazzaferro et al., 2013). For cytotoxic drugs, the new delivery systems can maintain or even increase the therapeutic efficacy and reduce toxic and side effects. Moreover, the targeted drug delivery system can accurately target tumor cells and improve bioavailability and selectivity, becoming one of the possible directions for cytotoxic drugs to be used for tumor treatment (Hitchcock et al., 2020).

Synthetic and natural polymers are primarily used as a carrier for drug delivery, including polylactic acid (PLA), lactic acid glycolic acid copolymer (PLGA), silk fibroin, chitosan, and corn protein (Estey et al., 2006; Pascoli et al., 2018). In general, natural polymer materials have good biocompatibility and biodegradability and are feasible to be a carrier for constructing nano-drug delivery systems (Mathur and Gupta, 2010; Mohammed et al., 2017). Silk fibroin, a medical material approved by the US Food and Drug Administration (FDA), has been widely used in the postoperative suture, tissue regeneration, and drug delivery system (Yucel et al., 2014). On the basis of the advantages of excellent mechanical properties and good biocompatibility and biodegradability, drug loaded nanoparticles can be prepared by using silk fibroin to acquire various biological functions (Qi et al., 2017). However, there are no anti-tumor nanomedicine with silk fibroin as drug carrier on the market yet. Currently, only albumin and silk fibroin are close in characteristics and categories among the anti-tumor nanomedicine approved for marketing, providing some reference and basis for the clinical transformation of silk fibroin in the future nanomedicine. Some of approved anti-tumor nanomedicines are shown in Table 1. Herein the structure, properties, applications and preparation of silk fibroin nanoparticles were introduced to get better understanding of silk fibroin and its application in anti-tumor drug delivery system. The research progress and achievements of silk fibroin as a carrier material of drug delivery system in tumor drug therapy in the past decade were also summarized and discussed. Finally, we further analyzed the current problems and future application prospects of silk fibroin and put forward some personal ideas.

**TABLE 1:** Some approved anti-tumor nanomedicines.

Types	Active pharmaceutic ingredient	Trademark	Types of tumor	First approval
Liposome	Doxorubicin	Doxil/Caelix	Ovarian cancer, metastatic breast cancer, Kaposi sarcoma	United States 1995
	Doxorubicin	Mycoet	Breast cancer	United States 1996
	Daunorubicin	DaunoXome	Kaposi sarcoma	United States 1996
	Leurocristine	Onco-TCS	Non-Hodgkin lymphoma	United States 1996
	Paclitaxel	Lipusu	Ovarian cancer, metastatic gastric cancer	China 2003
	Mifamurtide	MEPACT	Osteosarcoma	Europe 2009
	Vincristine	Marqibo	Leukemia	United States 2012
	Paclitaxe	PICN	Metastatic breast cancer	India 2014
	Irinotecan	Onivyde	Metastatic pancreatic cancer	United States 2015
				China 2022
	Paclitaxe	DHP107	Advanced gastric cancer	Korea 2016
	Cytarabine/daunorubicin	VYXEOS	Acute myeloid leukaemia	United States 2017
	Mitoxantrone	DuoEnDa	Peripheral T-cell lymphoma	China 2022
Polymer micelles	Paclitaxel	Genexol-PM	Breast cancer, lung cancer, pancreatic cancer	Korea 2006
	Docetaxel	Nanoxel M	Squamous cell carcinoma of head and neck	Korea 2012
	Paclitaxel	Paclical	Oophoroma	Russia 2015
	Paclitaxel	−	Non-small cell lung cancer	China 2021
Nanoparticles	Albumin-paclitaxel	Abraxane	Metastatic breast cancer	United States 2005
	Doxorubicin	Transdrug	Hepatocellular carcinoma	−
	−	NanoTherm	Recurrent glioblastoma	Europe 2010
	−	Hensify	Locally advanced soft tissue sarcoma	Europe 2019
Microsphere	90Y	SIR-Spheres	Metastatic colorectal cancer liver	China 2022
	Goserellin	−	Breast cancer	China 2022

## 2 Major characteristics of silk fibroin

### 2.1 The chemical structure

**FIGURE 1:**
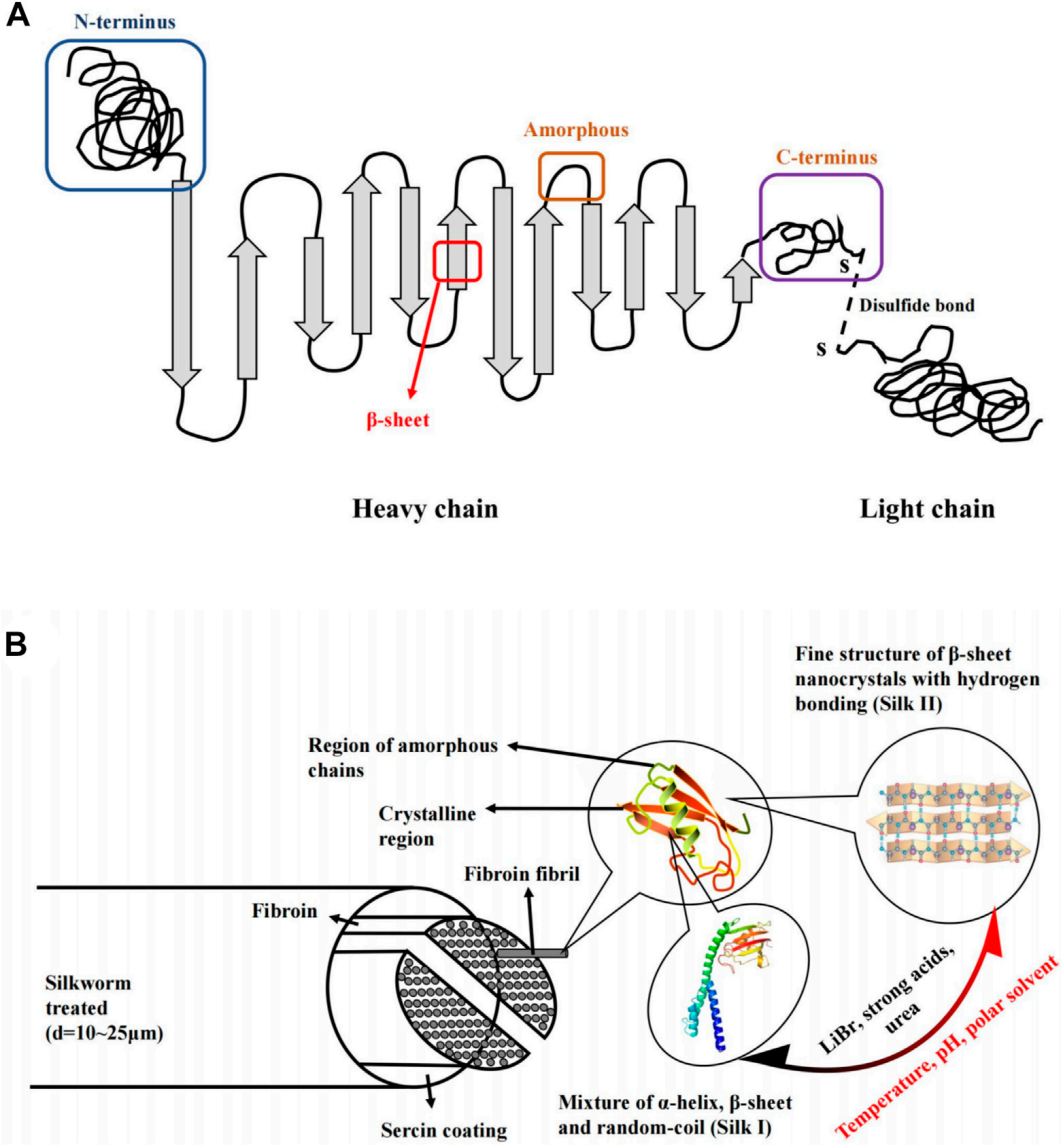
Schematic diagram of the silk structure. **(A)** heavy chain (i.e., N-terminus, β-sheets, Amorphous and C-terminus). and light chain which linked *via* disulphide bonds. **(B)** silkworm thread, fibril overall structure and silk fibroin polypeptide chains. Notes: Sun, W., Gregory, D.A., Tomeh, M.A., Zhao, X., 2021. Silk fibroin as a functional biomaterial for tissue engineering. International Journal of Molecular Sciences 22, 1499. Reprinted with permission from references. Copyright 2021, MDPI.

Silk fibroin is the main component of silk, accounting for about 75% of the total weight of silk. Silk fibroin consists of two leading chains, a heavy (H-) chain (390 kDa) and a light (L-) chain (26 kDa), which are linked by disulfide bonds to form the HL complex (Figure 1A) (Holland et al., 2019; Sun et al., 2021). P25 (25 kDa) is a glycoprotein, including ASN linked oligosaccharide chain, which is hydrophobically linked to H-L complex (Tanaka et al., 1999). Three structural protein subsets (heavy chain, light chain, and glycoprotein P25) comprise silk fibroin with a molecular ratio of 6:6:1 (Koh et al., 2015). Among them, the H- chain of silk fibroin has amphiphilic properties, including hydrophobic and hydrophilic blocks (Gholipourmalekabadi et al., 2020). The hydrophilic region is short and non-repetitive, but the hydrophobic region has repetitive sequences. It can be folded into a β-sheet to generate a crystal structure, so silk fibroin can be considered a hydrophobic glycoprotein and is insoluble in water (Bini et al., 2004).

Silk fibroin has two main crystal structures, including silk I and silk II (Figure 1B). Silk I is a metastable crystal structure containing bound water molecules and is intermediate between α-helix and β-fold. Silk I is hydrophilic and thermodynamically unstable. Silk II is the most stable state and is an antiparallel β-folded lamellar structure due to strong hydrogen bonds between adjacent peptide blocks, resulting in increased mechanical properties, including stiffness and tensile strength (Sun et al., 2021). On the contrary, silk II is hydrophobic and thermodynamically stable. Besides, the water solubility of silk fibroin was significantly related to the crystalline state and environmental pH value. Because silk I is more soluble than silk II, and the methods of transforming insoluble silk II into silk I can increase the water solubility of silk fibroin (Pham and Tiyaboonchai, 2020). Similarly, silk I can also be converted into silk II by changing solution conditions and ambient temperatures, such as temperature, pH, methanol, and potassium chloride, which can induce this conversion (Rusa et al., 2005; Chen et al., 2007; Huot et al., 2015; Ming et al., 2015; Gregory et al., 2016). Due to the role of hydrogen bonds, it is relatively challenging to convert silk II to silk I, and it can only be converted after LiBr, strong acid or urea is used to break the hydrogen bonds (Chen et al., 2007; Liu et al., 2011; Zheng et al., 2016). The theoretical isoelectric point pI of silk fibroin is 4.53. When the environmental pH is near the isoelectric point, the water solubility of silk fibroin will decrease (Lammel et al., 2010). Based on silk fibroin’s water solubility, organic solvents can be reduced during the preparation, thus reducing the impact on the encapsulated drugs. Nowadays, this technique is widely used for biomaterial engineering applications, including the silk fibroin nanoparticles based on transformation from silk I with a random coil and α-helix structure to the highly ordered β-sheet structure of silk II (Ma et al., 2020). Moreover, the regenerated silk fibroin solution has a small amount of unstable silk III in the air/water interface, which is a triple helical crystal structure (Valluzzi et al., 1999; Pham et al., 2019).

### 2.2 Biocompatibility and safety

As a kind of natural fiber, the medical use of silk as a wound suture can be traced back hundreds of years. Nowadays, biocompatible and safety are the main concern for silk fibroin used as a drug delivery carrier. Degummed silk and regenerated silk fibroin are considered biological materials suitable for animal cell culture, which has been confirmed for a long time (Minoura et al., 1995; Lazaris et al., 2002). Wang et al. used a vacuum ultraviolet-ozone activation method to wrap silk fibroin on the surface of MgZnCa alloy as a protective barrier and then implanted it in cells and rabbits to evaluate the corrosion resistance and biocompatibility of MgZnCa alloy (Wang et al., 2019a). The tests showed that this silk fibroin-coated MgZnCa had improved biocompatibility with bone marrow mesenchymal stem cells. According to another study about the stents made of silk implanted in mice, almost no inflammation and immune response were found after 1 year of feeding (Wang et al., 2008). Silk fibroin is a natural high-purity protein secreted and synthesized by endothelial cells on the inner wall of silkworm silk gland. The body can absorb its final degradation product, and its molecular weight can also be adjusted by modifying the composition of silk fibroin to meet the requirements of different biological environments (Guo et al., 2021). Therefore, it has good biocompatibility.

Some researchers have discussed the safety and effectiveness of microparticle scaffold prepared from silk fibroin for bone void filling (Deshpande et al., 2022). *In vitro* and *vivo* studies showed that this scaffold was biocompatible through cytotoxicity, immunogenicity, genotoxicity, systemic toxicity, and implantation. At the same time, compared with commercial calcium-based bone gap fillers, microparticle scaffold prepared from silk fibroin is non-toxic for bone gap filling and contributes to the good healing of fracture defects. In addition, the effectiveness and safety of engineered silk fibroin and collagen tissue have been evaluated in patients with bone defects. The results showed that it is safe and effective and suggested that a larger sample of randomized controlled trials can be carried out in the next stage (Sharafat-Vaziri et al., 2020). The latest study reported that inhalable ciprofloxacin hydrochloride microparticles (CMs) based on silk fibroin and mannitol (Lin et al., 2021). A series of *in vitro* and *in vivo* performance evaluation tests demonstrated that CMs did not affect lung function nor induce the secretion of inflammatory cytokines in the lungs. CMs, thus would be a promising pulmonary drug delivery system for the treatment of non-cystic fibrosis bronchiectasis. All these findings revealed that silk fibroin is a kind of natural polymer material that has a prominent advantage in the application of drug carriers due to its excellent biocompatibility and biosafety.

### 2.3 Mechanical properties and biodegradability

Four secondary structures of silk fibroin exist, including β-sheet, random coil, α-helix, and β-turn (Choi et al., 2018). Among them, the β-sheet structure in the crystalline region of silk fibroin is the main factor that plays a vital role in the excellent flexibility and tensile resistance of silk, and the toughness is higher than other synthetic fibers, such as carbon fiber, collagen (Gosline et al., 1999; Cheng et al., 2014; Choi et al., 2018; Blamires et al., 2020). Although natural silk fibroin has good mechanical properties, the biological materials made of silk fibroin, such as scaffolds, are made by regenerating silk fibroin solution. However, these scaffolds are highly fragile because the regenerated silk fibroin lacks appropriate secondary and hierarchical structures (Altman et al., 2002). In order to deal with the problem of poor mechanical properties of regenerated silk fibroin, some technology methods are used to manipulate the regeneration process to improve its mechanical capabilities, such as porogen and 3D bioprinting technology (Nazarov et al., 2004; Floren et al., 2016).

When exogenous substances enter the body, they generally cause immunogenic reactions. However, after degumming, silk fibroin does not contain sericin, so it will not produce immunogenicity (Florczak et al., 2021). Some studies have found that pure sericin can be used as a material with good biocompatibility, so the coexistence of silk fibroin and sericin may lead to an immune response (Chirila et al., 2013). On the contrary, silk fibroin is a polymer that enzymes can degrade, and its degradation products also do not have immunogenicity (Cao and Wang, 2009). Although these views are not consistent, they allow people to achieve different purposes of use by regulating the structure and performance of silk fibroin. It can be degraded by enzymes such as α-chymotrypsin, protease XIV and collagenase IA (Sun et al., 2021). Furthermore, its degradation rate is affected by the material’s microstructure, and the degradation rate is the random structure part, silk I structure, and silk II structure in the material from fast to slow (Umuhoza et al., 2020). Lee et al. investigated the degradation behavior of silk fibroin membranes *in vitro* and *in vivo*. They found that they were degraded in phosphate-buffered saline, medium and proteinase K solution (Lee et al., 2012). In protease K solution, 80% of this membrane was degraded within 10 days. Meanwhile, another study found also confirmed that, more importantly, its degradation products were also non-toxic and harmless (Lu et al., 2011). Some scholars synthesized silk fibroin hydrogels through a chemical cross-linking reaction of silk fibroin (Wu et al., 2020). By γ-ray irradiation at different doses, the chemically cross-linked silk fibroin exhibited better elasticity and better biodegradability and biocompatibility than the logistically cross-linked hydrogels. In the assay of cell proliferation, the study also evaluated the cell viability of human mesenchymal stem cells on freeze-dried silk fibroin hydrogel scaffolds. No significant cytotoxicity to human mesenchymal stem cells was observed.

## 3 Applications in drug delivery system

Due to the unique properties mentioned above, silk fibroin has been widely used as carrier material for various drug delivery systems. In fact, silk fibroin is usually prepared into diverse structural forms with disparate properties to adapt to various drug delivery systems, including hydrogels, porous scaffolds, rods, fibers, and nanoparticles (Zhao et al., 2015). Meanwhile, the drugs loaded could be the first-line drugs used in the clinic such as doxorubicin, and dexamethasone sodium, as well as some Chinese medicine monomers such as quercetin, curcumin (Kim et al., 2014). Since carrier forms of silk fibroin are various, the objects of action and diseases are also different. Therefore, the application of more detailed structural types of silk fibroin is shown in Table 2.

**TABLE 2:** The application of silk fibroin as drug carrier.

Delivery system	Loaded drug	Other excipients	Application	Subject	References
Porous scaffolds	−	TiO_2_	Tissue engineering	Mice and NIH 3T3 cells	Kim et al. (2014)
Discs	Griffithsin	−	HIV	Rhesus macaques	Crakes et al. (2020)
Films	Inactivated polio vaccine (IPV)	−	Vaccine	Wistar rats	Stinson et al. (2020)
Porous scaffolds	Irisin	Calcium silicate, sodium alginate	Bone regeneration	Bone marrow stem cells and SD rats	Xin et al. (2019)
Hydrogels	Betamethasone	Tyramine-gellan gum	Rheumatoid arthritis	New Zealand white rabbits and chondrogenic primary cells	Oliveira et al. (2020)
Porous scaffolds	−	Sodium alginate, polyvinyl alcohol, magnesium hydroxide	Antibiosis	Hu02 cells	Eivazzadeh-Keihan et al. (2020)
Hydrogels	Dexamethasone sodium phosphate	Chitosan	−	L929 fibroblast cells	Akrami-Hasan-Kohal et al. (2021)
Porous scaffolds	Quercetin	Hydroxyapatite	Osteogenesis	Bone marrow-derived mesenchymal stem cells and SD rats	Song et al. (2018)
liposome	Basic fibroblast growth factor	Liposome	Deep second-degree scald	Mice and NIH/3T3 fibroblast cells	Xu et al. (2017)
Nanoparticle	Celecoxib or curcumin	−	Osteoarthritis	Human articular chondrocytes	Crivelli et al. (2019)
Films	−	Poly ethylene glycol (PEG)	Limbal stem cell deficiency (LSCD)	Limbal epithelial stem cells and rabbits	Li et al. (2017)
Porous scaffolds	Dental pulp stem cells	Hydroxyapatite	Dental pulp regeneration	Nude mice	Zhang et al. (2019)
Liposome	Doxorubicin	Liposome	Cancer	MCF-7 cells, MDA-MB231 cells and L929 fibroblasts	Suyamud et al. (2021)
Scaffolds	Stromal cell derived factor-1 (SDF-1) and bone morphogenetic protein-2	Nano-hydroxyapatite	Bone regeneration	Bone marrow mesenchymal stem cells	Shen et al. (2016)
Films	An antimicrobial peptide (Cys-KR12)	−	Wound healing	Monocytes (Raw264.7)	Song et al. (2016)
Hydrogels	−	Bioink, graphene oxide	Tissue engineering	−	Kim et al. (2021)
Microneedle	Composite insulin	-	Diabetes	Mice	Zhu et al. (2020)
Microneedle	Melatonin	Proline, melatonin	Insomnia	Rats	Qi et al. (2021)
Scaffolds	−	Gelatin	Bone tissue engineering	Rats and mesenchymal stem cell	Luetchford et al. (2020)
Microcapsules	Plasmid DNA	−	−	NIH 3T3 cell and mice	Li et al. (2014)
Scaffolds	Usnic acid	−	Wound healing	Mice	Zha et al. (2021)
Nanofiber	Cationic peptide	Chitosan	Wound healing	Hu02 human foreskin fibroblast cells	Khosravimelal et al. (2021)
Discs	Curcumin	Poly(lactic acid)	Cancer	human cervical cancer cells	Patwa et al. (2018)

Nanoparticles based on silk fibroin are the most widely used and studied due to the simple and diverse processing technics and the improved *in vivo* ADME properties steming from the ideal size. The small size usually allows nanoparticles to penetrate tiny capillaries, thereby enhancing the cellular uptake of encapsulated therapeutic molecules (Mathur and Gupta, 2010; Zhao et al., 2015). Moreover, silk fibroin-based nanoparticles may deliver anti-cancer agents to tumor sites and thus have great potential for targeted drug delivery systems. Farokhi et al. also discussed the application of silk fibroin as electrospun nanofibers in the drug delivery system. For example, antibiotics, vitamins, and growth factors have been delivered using silk fibroin electrospun nanofibers as carrier materials and achieved remarkable results (Farokhi et al., 2020). In recent years, silk fibroin as a drug delivery carrier material has made significant development in the field of medicine, including HIV (Crakes et al., 2020), vaccine (Stinson et al., 2020), bone regeneration (Xin et al., 2019), rheumatoid arthritis (Oliveira et al., 2020).

We know that nanoparticles are a common structural form. As a new biomaterial, it is more and more widely used in the medical field. It has significant advantages, such as prolonging the half-life of drugs, reducing the dosage and frequency of administration, enhancing the curative effect, reducing the toxicity of drugs, increasing the accumulation of drugs in tissues, and realizing diversified routes of Administration (Chen et al., 2017; Hu et al., 2020a; Zhao et al., 2021). The preparation of silk fibroin nanoparticles has become a current hotspot. The desolvation method is the most common method to prepare silk fibroin drug-loaded nanoparticles. It is the process of spontaneously forming insoluble nanoparticles after mixing an aqueous silk fibroin solution with a water-miscible organic solvent. At the same time, in order to meet the requirements of silk fibroin nanoparticles for different purposes, most drugs are connected with silk fibroin through physical adsorption or chemical crosslinking to prepare nanoparticles (Montalbán et al., 2018). However, chemical modification is required to connect silk fibroin with other carrier materials when preparing composite carriers before drug loading.

Zhang et al. added 5.0% regenerated silk fibroin solution into water-miscible organic solvents such as acetone and ethanol, the organic solvent volume accounted for at least 70% of the final volume, and silk fibroin was suspended in the mixture of water and organic solvent to form Tiny spherical particles with good crystallinity, nanoparticles spontaneously aggregate to produce slow sedimentation, and finally the sediment can be collected and purified from the mixture by centrifugation and filtration to obtain particles with a particle size ranging from 35 to 125 nm (Zhang et al., 2007). Some scholars used electrospraying to prepare silk fibroin nanoparticles and connected them to the syringe through the nozzle of the high-voltage electrostatic generator, and fixed the whole assembly to the microinjection pump (Qu et al., 2014). Silk fibroin solution was differentiated into droplets under a high-voltage electrostatic field, and then the resulting droplets were continuously collected and frozen in liquid nitrogen. Subsequently, it was freeze-dried in a lyophilizer for 48 h. In order to separate the nano-sized particles, the lyophilized products were suspended in deionized water, then centrifuged, and lyophilized again to obtain silk fibroin nanoparticles (59–75 nm). In addition, we also summarize and describe other standard preparation methods, and their advantages and disadvantages are also shown in Table 3. In these methods, we also find that the size of silk fibroin nanoparticles ranges from 20 nm to 20 μm (Florczak et al., 2021).

**TABLE 3:** Common preparation methods for nanoparticles of silk fibroin.

Methods	Advantages	Disadvantages	Particle size	References
Desolvation	Mild conditions; simple operation; controllable volume	Particles are easy to polymerize; low drug retention and loading capacity; organic solvents are not easy to elute; cumbersome washing steps	35–125 nm	Zhang et al. (2007)
Electrospraying	No organic solvent; size controllable nanoparticles; high drug loading and encapsulation efficiency; high purity and excellent monodispersity	High cost; complex operation	59–75 nm	Qu et al. (2014)
Spray-freeze-drying or Spray-drying	No organic solvent; size controllable nanoparticles; uniform appearance; high drug loading and encapsulation efficiency	Freeze drying agent is required; high cost	∼5 μm	Kim et al. (2015)
PVA blend film	Mild conditions; simple operation; no organic solvent	Polymer residues; silk II should be induced after post treatment	300 nm–20 μm	Wang et al. (2010)
Supercritical fluid	No organic solvent; size controllable nanoparticles; high drug loading and encapsulation efficiency	High cost; complex operation	∼50 nm	Zhao et al. (2012)
Laminar jet break-up	No organic solvent; mild conditions; high drug loading and encapsulation efficiency	Big particle size; silk II should be induced after post treatment	30–440 μm	Wenk et al. (2008)
Capillary microdot	Nanoparticles are small and controllable	Silk II should be induced after post treatment	20–130 nm	Gupta et al. (2009)
Microemulsion	Nanoparticle size controllable	Residual organic solvent	167–169 nm	Myung et al. (2020)
Ball milling	Simple operation; easy to scale up	Wide size distribution; grinding impurities; May reduce silk II content	0.2–4 μm	Rajkhowa et al. (2008)
Salting out	Simple operation; high yield; no organic solvent; sustainable protein activity	Hydrophobic drugs are not easy to be embedded; salting-out agent residue; complicated washing steps	486 nm–2 μm	Lammel et al. (2010)
Crosslinking reaction	Simple operation; controllable volume	Complex operation; residual organic solvent	0.3–1 μm	Pham et al. (2019)

## 4 Drug delivery system for chemotherapy of silk fibroin

As mentioned above, the recent main antitumor treatment methods mainly include surgery, radiotherapy, chemotherapy, immunotherapy, etc. Each method has its advantages and disadvantages (Gao et al., 2014; Gao and Lo, 2018; Kaushik et al., 2019; Luo et al., 2019; Boreel et al., 2021). Nevertheless, most patients choose drugs for tumor treatment, such as cytotoxic drugs, but some patients also choose targeted drugs, traditional Chinese medicine decoction, and so on (Jiang et al., 2021; Spiliopoulou et al., 2021; Cortés et al., 2022). However, many anticancer drugs potentially cause toxicity in patients, resulting in high drug resistance and poor prognosis quality of life (Chen et al., 2016; Bakos et al., 2018; Messina et al., 2019; Abravan et al., 2020). A novel cancer therapy strategy is urgently needed. Drug delivery systems are becoming hotspots since they could reduce adverse reactions, and improve efficacy and safety, especially in cytotoxic drugs. There seem two primary reasons. First, the advent of albumin-paclitaxel, paclitaxel is a cytotoxic drug, and albumin-paclitaxel as a nano-drug has been successfully marketed and used in clinical practice, which has extensively promoted the development and progress of drug delivery systems (Lv et al., 2021). Secondly, cytotoxic drugs have serious side effects, and some drugs even have problems such as poor water solubility and short half-life, which limit their application (Newell and Sausville, 2016). In order to further solve the use of cytotoxic drugs during chemotherapy, the application of biomaterials in drug delivery systems is an emerging technology (Mancipe Castro et al., 2021). Drug delivery systems prepared by loading cytotoxic drugs with biomaterials can reduce these problems, improve their therapeutic effects, and expand their clinical applications (Al-Mansoori et al., 2021). Therefore, we believe that cytotoxic drugs will gradually become the focus of drug delivery systems. Additionally, drug carrier materials are a determinant in delivery systems. PLA or PLGA was used to build drug delivery systems in many studies and approved by FDA for drug injection. Although various nanocarrier systems are available in the market, a gap exists in drug delivery systems based on different carriers (Aggarwal et al., 2006).

Nanotechnology can be applied to the delivery of drugs, providing a new way of cancer treatment. In the past decade, it has been found that materials that can be used as drug carriers include polyurethane functionalized starch (Desai et al., 2021), ferritin(He et al., 2019), gold nanoparticles (AuNPs) (Li et al., 2019), cyclodextrin (Kang et al., 2019), and silk fibroin (Kapoor and Kundu, 2016; Melke et al., 2016). Among them, silk fibroin, a typical natural biomaterial, exhibits versatile properties through tuning the secondary conformations and hierarchical microstructures (Ma et al., 2020). Silk fibroin can be prepared into various drug delivery systems to meet the different requirements of cancer treatment (Wani and Veerabhadrappa, 2018; Xu et al., 2019; Zuluaga-Vélez et al., 2021). In addition, it also has shown promising potential as a carrier in tissue repair (Gholipourmalekabadi et al., 2020), bone defects (Hong et al., 2020a), myocardial infarction (Chen et al., 2018b), nerve repair (Fernández-García et al., 2016), colitis (Diez-Echave et al., 2021), infections (Hassani Besheli et al., 2017), acute pancreatitis (Hassanzadeh et al., 2021), osteoporosis (Wang et al., 2018), and cancers (Xie et al., 2016). At present, silk fibroin has been studied and analyzed in the field of tumor as a carrier material for drug delivery.

### 4.1 Breast cancer

**FIGURE 2:**
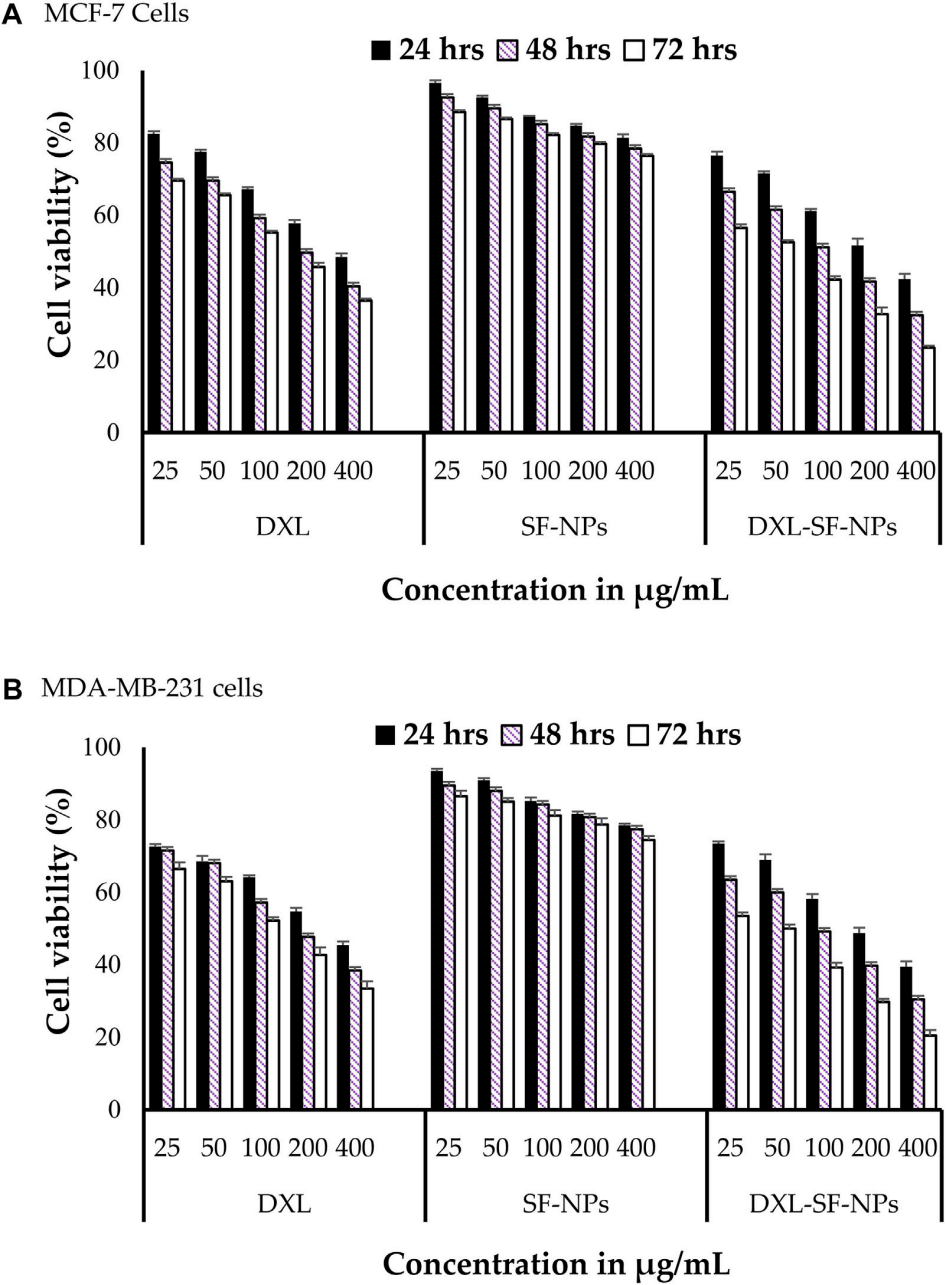
*In vitro* cytotoxicity of DXL-loaded SF-NPs against breast cancer cell lines. **(A)** MCF-7 and **(B)** MDAMB-231 cells were incubated with different concentrations of free DXL, SF-NPs, or DXL-loaded SF-NPs for different incubation times Notes: Al Saqr, A., Wani, S.U.D., Gangadharappa, H.V., Aldawsari, M.F., Khafagy, E.S., Lila, A.S.A., 2021. Enhanced cytotoxic activity of docetaxel-loaded silk fibroin nanoparticles against breast cancer cells. Polymers 13: 1416. Reprinted with permission from references. Copyright 2021, MDPI.

Docetaxel can be used to treat breast cancer. However, low water solubility and systemic toxicity seriously hinder its clinical application. Researchers prepared docetaxel-loaded silk fibroin nanoparticles and analyzed their potential antitumor activity against breast cancer cell lines (Al Saqr et al., 2021). The diameter of these nanoparticles was 178–198 nm, with net negative surface charge and encapsulation efficiency ranging from 56% to 72%. *In vitro* release study showed that the nano-formulation had a biphasic release curve and could release the drug for 72 h. *In vitro* cell study revealed that compared with free drugs, the nano-formulation significantly increased the cytotoxic potential of breast cancer cell lines and enhanced the uptake of docetaxel by breast cancer cells (Figure 2). Furthermore, the accumulation of cells treated with nano-formulation in the G2/M phase was significantly higher than that of cells treated with free docetaxel. Compared with free docetaxel, the nano-formulation loaded with docetaxel has excellent antitumor activity on breast cancer cells. The results also emphasized the feasibility of using silk fibroin-made nanoparticles as a non-toxic and biocompatible delivery carrier to enhance breast cancer treatment. At the same time, silk fibroin has good biocompatibility and can be used as composite materials with other biological materials for drug delivery systems and targeted formulation development. *Arumugam et al.* made silk fibroin, cellulose acetate, and gold and silver nanoparticles (CA/SF/Au-Ag) into composite nanofibers. They explored their toxicity to MCF-7 and MDA-MB-231 human breast cancer cells (Arumugam et al., 2021). The study found that CA/SF/Au-Ag nanoparticles have a high inhibitory effect on breast cancer cells, with an IC50 value of 17.54 μg/ml. It also indicated that the targeted nanoparticles were relatively highly inhibitory on breast cancer cells with a lower IC50 than the other three groups. In the activity assay on MCF-7 and MDA-MB-231 cell lines, CA/SF/Au-Ag composite nanofibers exhibited excellent bioactivity compared with other groups. In total, this active targeting nanofiber has a high inhibitory effect on tumor cells with a low IC50. However, due to the lack of *in vivo* studies, it is difficult to determine the composite nanofibers’ active targeting and *in vivo* antitumor efficacy. Contemporarily, it has been used to build a nano-formulation loading rosmarinic acid, and the activity on breast cancer and cervical cancer cell lines was investigated (Fuster et al., 2021). The results showed that the nano-formulation could inhibit cell proliferation and induce apoptosis of HeLa and MCF-7 cell lines. Consequently, based on the above results, a silk fibroin-based nanoparticle delivery system can enhance the antitumor potential of anti-breast cancer drugs.

### 4.2 Liver cancer

**FIGURE 3:**
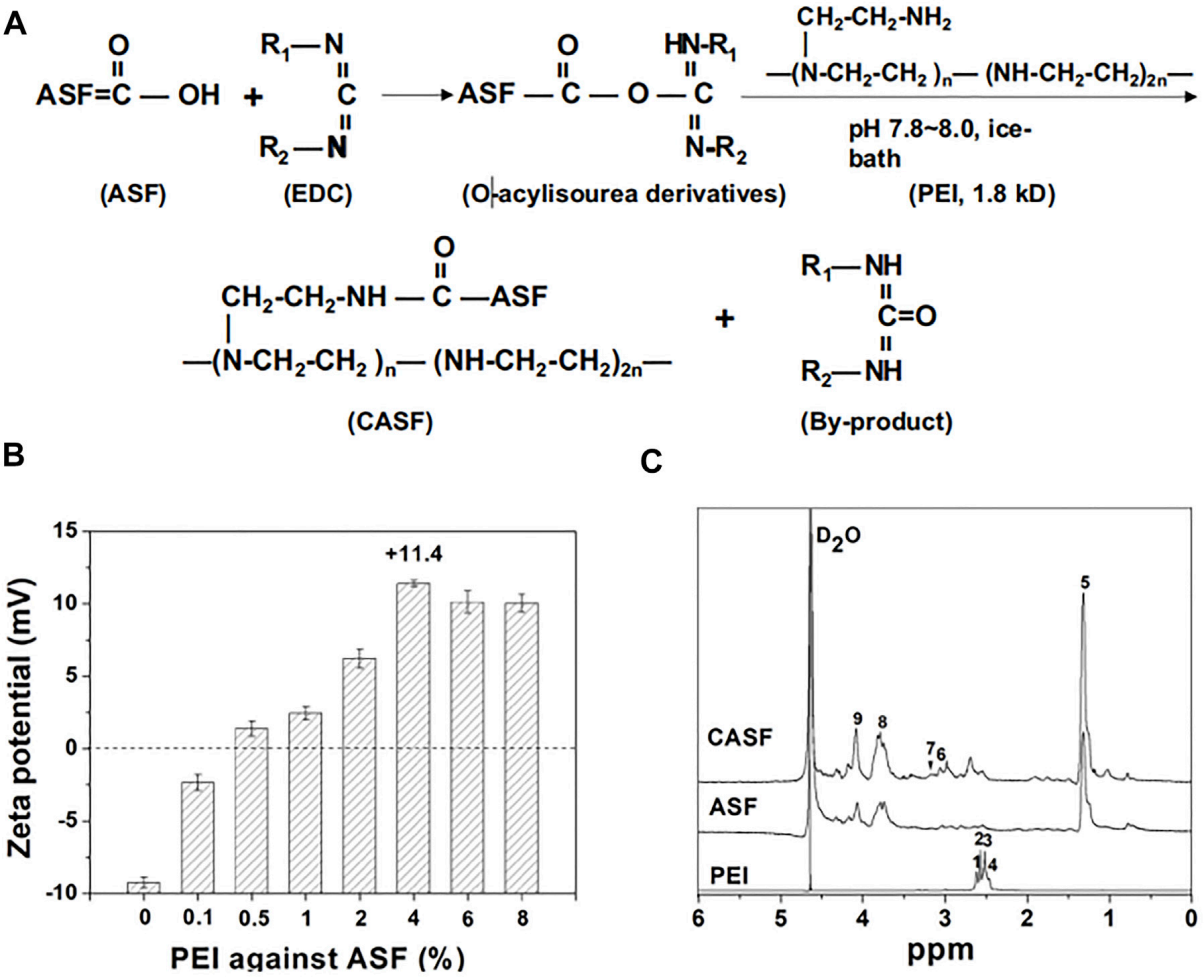
Synthesis and characteristics of CASF. **(A)** Schematic diagram of CASF synthesis. **(B)** Zeta potential of CASF at PEI/ASF weight ratios of 0%, 0.1%, 0.5%, 1%, 2%, 4%, 6%, and 8%. **(C)** 1 H-NMR spectra of PEI, ASF and CASF in D2O. Abbreviations: CASF, cationic *Antheraea pernyi* silk fibroin; PEI, polyethylenimine; ASF, *Antheraea pernyi* silk fifibroin; NMR, nuclear magnetic resonance. Notes: Qu, J., Wang, W., Feng, Y., Niu, L., Li, M., Yang, J., Xie, Y., 2019. Cationic *Antheraea pernyi* silk fibroin-modified adenovirus-mediated ING4 and IL-24 dual gene coexpression vector suppresses the growth of hepatoma carcinoma cells. International Journal of NanoMedicine 2019:14 9745-9761' Originally published by and used with permission from Dove Medical Press Ltd.

Transcatheter arterial chemoembolization is an effective method for the treatment of hepatocellular carcinoma. Some scholars successfully prepared sodium alginate-modified silk fibroin microsphere embolic agent by emulsion crosslinking method, loaded with adriamycin hydrochloride, and studied its toxicity to liver cancer cells (Chen et al., 2020). The results showed that: firstly, the microspheres had smooth surfaces as well as good sphericity, and the swelling rate of the microspheres could meet the requirements of an arterial embolic agent, also included the sensitivity to pH and temperature; secondly, the microspheres had good blood compatibility; thirdly, cytotoxicity test showed that microspheres could promote the proliferation of fibroblasts and HUVEC; Finally, the drug loading and release ability of the microspheres were proved by the controlled release curve of adriamycin hydrochloride. This study showed that sodium alginate-modified silk fibroin microspheres could be a potential biodegradable arterial embolization agent to treat liver cancer. Some scholars also successfully prepared the complex of silk fibroin and adenovirus by taking advantage of the excellent biocompatibility of silk fibroin, loaded growth inhibitor 4 and interleukin 24, and analyzed its effect on liver cancer cells (Qu et al., 2019). The preparation and characterization of this complex are shown in Figure 3. The results showed that the constructed complex could effectively mediate the expression of target gene ING4 in SMMC-7721 cells and the secretion of target gene IL-24 in SMMC-7721 cells, thereby inducing apoptosis of hepatoma SMMC-7721 cells. Besides, the viability of SMMC-7721 and L-02 cells infected with the complex was also assessed. It was found that the growth of SMMC-7721 cells was significantly inhibited but that the growth and proliferation of L-02 cells were not affected. The complex constructed in this study showed improved infection efficiency and enhanced suppressive effects on human hepatoma carcinoma SMMC-7721 cells, potentially reducing the dose of adenovirus and maintaining high infection efficiency and tumor inhibition. Currently, most researchers tend to make silk fibroin and other biological materials into a complex and then load drugs or others for anti-liver cancer research. The latest research on silk fibroin might be a drug delivery carrier material for liver cancer.

### 4.3 Lung cancer

Lung cancer is the most common cancer in terms of morbidity and death, thus choosing the right therapy is crucial (Mottaghitalab et al., 2015). In recent years, silk fibroin as a carrier material to load anti-cancer drugs to treat lung cancer has also become a hot research direction. In 2014, Qu et al. used silk fibroin loaded with cisplatin to prepare a controlled-release preparation to conduct research on lung cancer cell toxicity (Qu et al., 2014). It was found that cisplatin in the nanoparticles could be released slowly and continuously for more than 15 days. Meanwhile, *in vitro,* anti-cancer experiments showed that silk fibroin-nanoparticles loaded with cisplatin were easily internalized by A549 lung cancer cells, transferred cisplatin to cancer cells, and then triggered apoptosis. However, these particles were not easy to be internalized by L929 mouse fibroblasts, so their inhibitory effect on the growth of normal cells was weak. The silk fibroin nanoparticles loaded with cisplatin showed a sustained and effective killing effect on tumor cells, but the inhibitory effect on normal cells was weak. This research provided a new method for preparing cisplatin-loaded silk fibroin nanoparticles and offered a new carrier system for the clinical treatment of lung cancer and other tumors. *Mottaghitalab et al.* also used silk fibroin as a drug carrier to load gemcitabine into a targeted nano-formulation. They explored its therapeutic effect on lung cancer in the rat model (Mottaghitalab et al., 2017). Compared with the untargeted silk fibroin nano-formulation and the control group, the gemcitabine-targeted nano-formulation showed higher cytotoxicity, cell uptake and accumulation in lung tissue. Besides, nano-formulation targeting gemcitabine had higher potential in the treatment of induced lung tumors. In histological and radiological analysis, higher survival, lower mortality and no signs of metastasis were also observed in those animals treated with targeted agents. This study proposes an effective anti-cancer drug delivery system for targeted lung cancer. As its loaded drug, gemcitabine is limited in clinical use because it is cytotoxic. The preparation of nano-targeted formulation with silk fibroin helps to expand its use, improve the therapeutic effect, and reduce its toxic and side effects. The system may be used in the treatment of malignant lung cancer in the future. Moreover, this actively targeted nano-formulation shows a higher therapeutic effect, more aggregation effect and lower mortality in the study. This is a characteristic and advantage that untargeted nano-formulation does not have, and it also shows the advantages and potential of actively targeted nano-formulation. More interestingly, *Wang et al.* even found that silk fibroin peptide had a significant inhibitory effect on H460 lung cancer cells, induced the cells to arrest in the S phase, and then promoted apoptosis (Wang et al., 2019b). Silk fibroin peptide may become a new type of therapeutic agent for lung cancer treatment.

### 4.4 Gastric cancer

**FIGURE 4:**
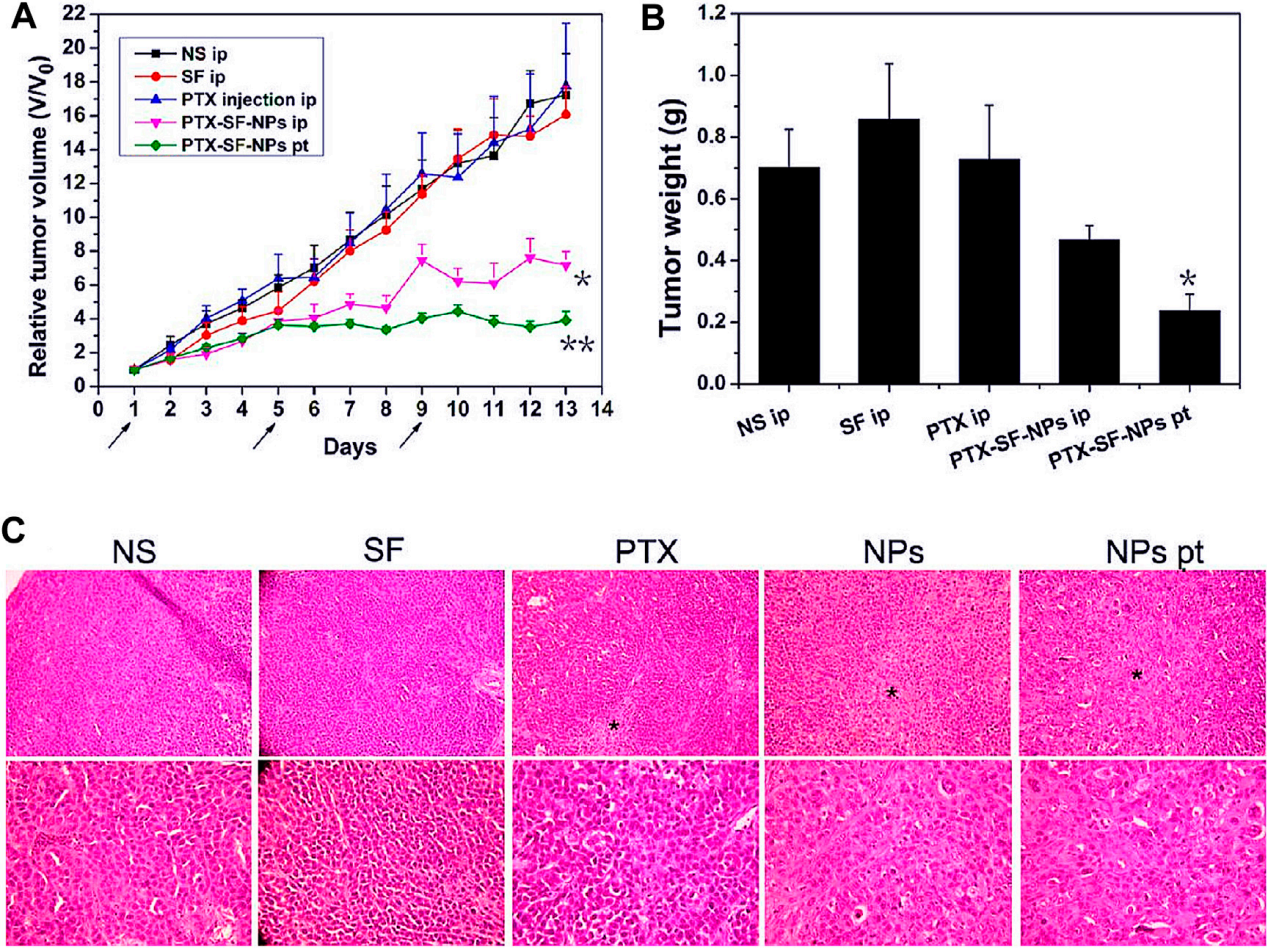
Antitumor efficacies of PTX-SF-NPs for systemic and focal treatment in human gastric cancer BGC-823 nude mice xenograft model. **(A)** Relative tumor volumes during treatment. **(B)** Absolute tumor weights on Day 14. **(C)** H&E staining of tumors Notes: Wu, P., Liu, Q., Li, R., Wang, J., Zhen, X., Yue, G., Wang, H., Cui, F., Wu, F., Yang, M., Qian, X., Yu, L., Jiang, X., Liu, B., 2013. Facile preparation of paclitaxel loaded silk fibroin nanoparticles for enhanced antitumor efficacy by locoregional drug delivery. ACS Applied Materials and Interfaces 5, 12638–12645 Reprinted with permission from references. Copyright 2013, American Chemical Society.

Silk fibroin has also been studied and analyzed in gastric cancer nano-formulations as a drug delivery carrier material. Some researchers prepared nano-formulation from silk fibroin and paclitaxel to explore their effects on human gastric cancer cell lines BGC-823 and SGC-7901 (Wu et al., 2013). *In vitro* cytotoxicity study, when paclitaxel was incorporated into the nano-formulation, paclitaxel could maintain its pharmacological activity, and silk fibroin had no cytotoxicity to cells. The antitumor effect of paclitaxel silk fibroin nano-formulation *in vivo* was evaluated on the transplanted tumor model of gastric cancer in nude mice. Compared with systemic administration, local administration of paclitaxel silk fibroin nanoparticles showed excellent antitumor efficacy by delaying tumor growth and reducing tumor weight (Figure 4). In addition, the organs of rats treated with nanoparticles did not show apparent toxicity, indicating that silk fibroin nanoparticles were safe *in vivo*. Another researcher loaded 5-fluorouracil on the composite made of cyclic pentapeptide and silk fibroin to prepare targeted nanoparticles and investigated its tumor progress on the rat model of gastric cancer (Mao et al., 2018). These results also clarified that silk fibroin-based nano-formulation had ideal active tumor-targeting properties and significantly reduced tumor burden. Crgdfk was conjugated with silk fibroin polypeptide to make the nanoparticles of 5-fluorouracil targeted. In this study, this preparation method makes the prepared nanoparticles have targeted characteristics, and 5-fluorouracil can reach more tumor cells to give full play to the antitumor effect. At the same time, the rat organs treated with silk fibroin nano preparation did not show apparent toxicity, which proved that it had good biocompatibility and safety *in vivo*. In this study, nano preparations with active targeting showed obvious advantages, and the efficacy was more effective when combined with photodynamic therapy. In 2020, Zhang et al. prepared silk fibroin into a hydrogel loaded with gambogic acid to prepare a nano-formulation and studied its antitumor activity in the animal model of gastric cancer (Zhang et al., 2020a). The results showed that the antitumor activity of the hydrogel combined with gambogic acid nano-formulations was higher than that of the hydrogel loaded with gambogic acid and free gambogic acid. The free gambogic acid group had almost no antitumor effect. There was no significant change in body weight in each group, and the white blood cell and hemoglobin counts decreased slightly. The hydrogel delivery system prepared by silk fibroin as a drug carrier showed little systemic toxicity. It was considered a new strategy to improve the efficacy of anti-gastric cancer.

### 4.5 Pancreatic cancer

Pancreatic cancer is a malignant tumor. Its survival rate is extremely low, and its prognosis is still very poor. Hence, novel treatment strategies to combat this deadly disease are urgently needed. Some researchers used silk fibroin as a carrier material to load triptolide and triptorubin to prepare nanoparticle preparations and evaluated its effect on human pancreatic cancer cells (Ding et al., 2017). The results showed that the silk fibroin nano-formulation of triptolide could produce a synergistic inhibitory effect on the growth of MIA PaCa-2 and PANC-1 cells, and the growth inhibitory effect of the combination of the two drugs is synergistic rather than additive. It also further suggested that this novel combination may provide a potential treatment for pancreatic cancer. However, there are few studies on silk fibroin related to pancreatic cancer. The effect of silk fibroin as a drug delivery carrier material in the treatment of pancreatic cancer needs further research to confirm. In order to further clarify the application of silk fibroin in drug delivery systems for tumor treatment, we summarized the details and results of silk fibroin as a carrier material for drug delivery systems in different tumor treatment research in the past decade (Table 4).

**TABLE 4:** The related research on silk fibroin as a carrier for drug delivery systems in cancer.

Delivery system	Anti-cancer agent	Other excipients	Size	Applica-tion	Major findings	References
Nanoparticle	Curcumin	−	155–170 nm	Cancer	↑ efficacy and cytotoxicity against neuroblastoma cells	Montalbán et al. (2018)
Nanoparticle	Doxorubicin	Tween-80	100–200 nm	Gliobla-stoma	↑ sustained drug release behavior of the loaded drug (doxorubicin)	Pandey et al. (2020)
Nanoparticle	Curcumin	−	<100 nm	Colon cancer	↑ anti-cancer efficacy nanoparticles was improved, ↓ cytotoxicity of normal cells at a concentration of ∼10 μg/ml	Xie et al. (2017)
Hydrogels	Salinomycin and paclitaxel	−	120 nm	Cancer	This might become a novel and powerful locoregional tumor treatment regimen in the future	Wu et al. (2018)
Porous scaffolds	Cisplatin	−	6 mm	Gastric cancer	↑ long-term efficacy and bioactivity of the injectable hydrogel system *in vitro* for sustained drug delivery application	Gangrade and Mandal, (2020)
Nanoparticle	Fingolimod	Heptapeptide, selenium	120 nm	Thyroid cancer	↑ permeability and retention of the tumor area; ↑ biocompatibility	Zou et al. (2021)
Nanoparticle	Doxorubicin	Indocyanine green, MnO_2_	139.6 nm	Cancer	↑ tumor inhibition. ↓ systemic toxicity, ↓ adverse effect	Yang et al. (2019)
Nanoparticle	Disulfiram	ZnO	259.36 nm	Breast cancer	↑ tumor targeting, ↑ half-life, ↑ retention time in the body	Zhao et al. (2020)
Nanoparticle	Indocyanine green	−	165.9 nm	Breast cancer and cervical cancer	↑ stability of the drug, ↑ affinity	Chen et al. (2018a)
Nanoparticle	5-fluorouracil	PEG	<50 nm	Colorec-tal cancer	↑ biocompatibility in the HT-29 cells	Hudiță et al. (2021)
Nanoparticle	Indocyanine green	Proanthocyanidins	120.1 nm	Glioma	↑ blood circulation time ↑ tissue distribution	ZhuGe et al. (2019)
Hydrogel	Biliverdin	−	−	Glioma	↑ retention time, also protects biliverdin from enzyme-mediated metabolism and ↑ its lifespan	Yao et al. (2020)
Nanoparticle	Doxorubicin	−	130 nm	Cancer	The magnetic tumor-targeting ability broadens the range of biomedical applications of silk fibroin	Tian et al. (2014)
Scaffolds	Doxorubicin	−	−	Breast cancer	This natural silk based 3D distribution system of cell culture can easily be exploited as high throughput screening system for cancer drug discovery and development	Subia et al. (2015)
Rods	Anastrozole	−	1.0–1.5 mm	Breast cancer	↑ viability of silk reservoir rod technology as a long-term sustained, near zero-order release dosage form	Yucel et al. (2014)
Nanoparticle	5-fluorouracil or curcumin	−	217 ± 0.4 nm	Breast cancer	Showing the future potential of nanoparticle-loaded binary drugs in the treatment of breast cancer	Li et al. (2016)
Fiber	Curcumin	−	<100 nm	Cancer	↑ intracellular uptake, ↑ anti-cancer effect, ↓ toxicity to normal cells	Xie et al. (2016)
Hydrogel	−	−	200 nm	Cancer	Displayed desired *in vivo* biocompatibility; these hydrogels would spontaneously acquire conformation change which allowed these hydrogels to serve as a biomimetic platform for modulation of encapsulated cell fate and suppression of cancer formation	Yan et al. (2016)
Nanoparticle	Cisplatin	Mannitol	5 μm	Lung cancer	Demonstrating cell compatibility with A549 human lung epithelial cell line	Kim et al. (2015)
Nanoparticle	Paclitaxel	iRGD-EGFR	18 ± 19.7 nm	Cancer	↑ tumor targeting, ↑ anti-cancer effect, ↓ tumor growth and tumor volumes	Bian et al. (2016)
Nanoparticle	Doxorubicin	Zeolitic imidazolate framework-8	220 nm	Breast cancer	↓ tumor growth, ↑ intracellular uptake, ↑ cell apoptosis	Chen et al. (2021)
Hydrogel	Iron oxide nanocubes	-	-	Hepatoc-ellular carcino-ma	The ferrimagnetic silk fibroin hydrogel with remote hyperthermia performance under an alternating magnetic field, resulting in the effective magnetic hyperthermia of deep tumors	Qian et al. (2020)
Nanoparticle	Alpha mangostin	-	300 nm	Cancer	↑ solubility, ↑ sustained release, ↓ hematotoxicity, ↑ anti-cancer effect	Pham et al. (2019)
Nanofiber	Diethyldithioc-arbamate	Polyethylene oxide	1,202 nm	Cancer	↑ biocompatibility, ↑ anti-cancer activity	El Fawal et al. (2021)
Nanoparticle	Gemcitabine	SP5-52 peptide	105–302 nm	Lung Cancer	↑ targeting, ↑ survival rate, ↓ mortality, no sign of metastasis	Mottaghitalab et al. (2017)
Nanoparticle	Tamoxifen	−	186.1 ± 5.9 nm	Breast cancer	Tamoxifen release from Nanoparticle exhibited a biphasic release profile with an initial burst release within the first 6 h and sustained release for 48 h, ↑ anti-cancer activity	Moin et al. (2021)

## 5 Prospects and conclusion

Over the past decade, silk fibroin has played an increasingly important role in drug delivery systems as a carrier material, which has aroused the extensive interest of researchers. As described in this article, silk fibroin-based nanocomplexes have been extensively studied in drug delivery and tumor therapy. Furthermore, the researchers have got some positive results in both *in vitro* and *in vivo* tests, a breakthrough in nanomedicine. By using cell or animal models, the nanocomposites prepared by silk fibroin as a carrier material have been studied in a variety of tumor diseases, such as breast cancer, liver cancer, lung cancer, stomach cancer, pancreatic cancer, colon cancer, thyroid cancer, cervical cancer, colorectal cancer, and glioma. The results have found that: 1) the use of fibroin nanoparticles as a non-toxic, biocompatible delivery carrier to enhance the feasibility of tumor treatment effects that have a little inhibitory effect on normal cells; 2) the fibroin can be prepared with other materials into a composite carrier (such as: cellulose acetate, gold and silver nanoparticles, adenovirus, gelatin, etc.), among them, the scaffold of silk fibroin loaded with amino acid modified adenovirus can stimulate the formation of vascular network and thus accelerate the regeneration of dermal tissue, and the composite nanofibers made of silk fibres have a high inhibitory effect on tumor cells (Wang et al., 2019c); 3) the surface of silk fibroin can be properly modified to meet different needs. For example, the sustained-release behavior of adriamycin can be enhanced by modifying the surface of silk fibroin with Tween 80 as surfactant; 4) silk fibroin can be prepared into nanocarriers with stimulation response, which can be triggered by the changes of pH value, ultraviolet, near infrared, ultrasonic and magnetic field, to accurately release the drug at the tumor site and enhance the curative effect; 5) the composite nanoparticles prepared from silk fibroin can also significantly improve the tumor inhibition effect through the combination of photothermal therapy (PTT)/tumor-specific photodynamic therapy (PDT)/chemotherapy, with minimal systemic toxicity or adverse reactions; 6) the combination with specifically targeted ligands (such as antibody, growth factor, transferrin, polypeptide, polysaccharide, etc.) helps to enhance the targeting of drug delivery, reduce the toxic and side effects of drugs, and strengthen tumor inhibitory effect in drug delivery system.

The targeted drug delivery system based on silk fibroin will significantly promote the treatment and development of tumors in the future, mainly due to the modifiability of the silk fibroin structure. The structure of it contains active amino and carboxyl groups, which researchers can chemically modify to increase the material’s functionality. It has certain biological activity, and SP5-52 peptide sequence (SVSVGMKPSPRP) can target the cell surface of non-small cell lung cancer (NSCLC) (Mottaghitalab et al., 2017). NSCLC cells can increase anticancer accumulation 5.7-fold in tumorigenic lung tissue compared to non-targeted vectors. The SP5-52 peptide was synthesized by solid-phase peptide synthesis on Rink amide MBHA resin. SP5-52 peptide was linked to the surface of silk fibroin nanoparticles with Hatu and diea as coupling reagents to obtain SP5-52 peptide modified nanoparticles. The results indicated that the silk fibroin nanoparticles targeting gemcitabine have a higher potential in treating induced lung tumors than the control group. The results of histopathology and radiological analysis showed that higher survival rate, lower mortality rate, and no signs of metastasis were also observed in those animals treated with SP5-52 peptide modified silk fibroin@gemcitabine. This study proposed an effective anticancer drug delivery system for specifically targeting induced lung tumors, which can be used to treat malignant lung cancer in the future. In addition to SP5-52 peptide, RGD tumor targeting peptide (Arginine-Glycine-Aspartate) (Rodriguez-Nogales et al., 2016), folic acid(Subia et al., 2014), chondroitin sulfate (Gou et al., 2019b), hyaluronic acid (Ziadlou et al., 2021) can modify silk fibroin for targeted delivery of drugs. Moreover, PEG can also modify silk fibroin, thereby extending the circulation time of drugs *in vivo* (Zhang et al., 2020b). Therefore, this kind of silk fibroin nano drug delivery system that actively targets tumors is also the focus of current and later research.

The application of silk fibroin in disease diagnosis, treatment and prevention has gradually developed, but it loaded anti-cancer drugs to prepare new drug delivery systems is a challenge for clinic use. Most cell and animal studies are short-term observations and lack long-term safety assessment, including survival analysis and toxic and side effects evaluation. In addition, the maximum allowable dose of silk fibroin, drugs, and other materials in animals seems to be an essential reference index for clinical use. Moreover, for the preparation of silk fibroin nanoparticles, whether the size and shape of particles will affect the distribution and accumulation of drugs in tumor tissues to affect the therapeutic effect remains to be further explored and analyzed. Fortunately, silk fibroin-related products such as Seri^®^, Surgical Scaffold, Surusil^®^, Sofsilk^TM^ and SickVoice^®^ are on the market, indicating that silk fibroin, as a new medical polymer material that has attracted much attention in recent years, has gone from basic research to clinical application. However, how to transfer the new drug delivery system based on silk fibroin loaded drugs from the laboratory to the clinic for further research and analysis, which first needs to be solved is the mass production of silk fibroin.

Although silk can be obtained through silkworm breeding, this production method has the problems of low yield, long time-consuming and low efficiency. Mehreen et al. believed that the cost of silk obtained from cocoon was low, which could reduce the overall production cost on a larger industrial scale, so as to obtain silk fibroin. However, this does not take into account other factors in the actual upload (Mehreen et al., 2021). At the same time, many reviews did not put forward views on the problems and solutions in the industrial production of silk fibroin, which is a key issue to promote the application of it (Florczak et al., 2014; Wani and Veerbhadrappa, 2018; Florczak et al., 2021; Chis et al., 2022). Based on this, this manuscript puts forward some views on this issue. For production enterprises, their production technology (such as the extraction, preparation, separation and purification of silk fibroin) and the quality control and batch release requirements in GMP mass production have great challenges. At the same time, there are problems such as product quality detection (how to detect the safety, biocompatibility and biodegradability of silk fibroin). These are urgent needs to be solved. Although the laboratory can extract and prepare silk fibroin by itself, few reports on the industrialized production of mature silk fibroin. There is no pharmacopoeia standard or influential industry standard in developed countries to include this variety. In addition, due to the heterogeneity of silk. There are significant differences in amino acid sequence, morphology and manufacturing process between the source materials; therefore, the results of their preclinical characteristics cannot be directly transformed between these studies (Florczak et al., 2020). Consequently, there may be a long time for the transformation, development, and commercialization of the silk fibroin-based drug delivery system. The fact that silk fibroin cannot be mass-produced and quality inspected is also the main reason why it has been difficult to achieve breakthroughs in drug delivery systems since it was approved by the FDA (Florczak et al., 2021). At present, in addition to obtaining silk fibroin from silk, spider silk can also be obtained by breeding spiders and further processed to obtain silk fibroin(Salehi et al., 2020). However, it is also difficult to industrialize and commercialize large-scale production, because spiders are carnivores, putting several spiders together will cause them to bite each other. They have a variety of silk glands, and different glands produce different silk properties (Rising and Johansson, 2015). Hence, it is difficult to collect silk with a single performance. At the same time, the secretion of spider silk from artificially reared spiders has dropped significantly, and the quality has also dropped significantly (Liu et al., 2021). There exist many ways to obtain spider silk, but the problem of quantity and quality is still difficult to solve properly, such as microbial pathway (Wu et al., 2017), animal pathway (Liebsch et al., 2018), plant pathway (Scheller et al., 2001) and electrospinning (Belbéoch et al., 2021). Therefore, the acquisition of silk fibroin is mainly obtained by breeding silkworms and harvesting cocoons.

However, we found that albumin is one of the preferred carrier materials for antitumor drug nanoparticles (Kunde and Wairkar, 2022). Albumin is a natural macromolecular material of protein. Human serum albumin has high stability, good biocompatibility and biodegradability, no immunogenicity and can be modified structurally (Hong et al., 2020b). It is worth noting that the fundamental properties of silk fibroin are similar to albumin, and it also has the internal stimulation response of pH/lysosomal enzyme/GSH/ROS. It has a wide range of sources, and the acquisition cost is much lower than that of albumin from human blood. Therefore, we believe that the successful experience of albumin as a drug carrier is worthy of reference and learning for silk fibroin. On the other hand, silk fibroin from different sources is different (Lehmann et al., 2022). The slow degradation of silk II crystal structure also makes silk fibroin unsuitable for the situation that silk fibroin needs to be quickly and thoroughly removed from the body, which may be an obstacle to the application of silk fibroin in drug delivery system (Hu et al., 2020b). Transgenic silk fibroin can achieve quality consistency (Gou et al., 2019a). It can also change the degradation rate of silk fibroin by adjusting the molecular structure. It has also been studied as a drug delivery carrier material, which is a promising and potential solution strategy (Xia et al., 2011; Hofer et al., 2012; Zheng and Zuo, 2021). When a safe and mass production method of silk fibroin is found and applied, the new anti-cancer drug delivery system mediated by silk fibroin will change greatly and be used in clinic.

## References

[B1] Abravan A. Faivre-Finn C. Kennedy J. McWilliam A. van Herk M. (2020). Radiotherapy-related lymphopenia affects overall survival in patients with lung cancer. J. Thorac. Oncol. 15, 1624–1635. 10.1016/j.jtho.2020.06.008 32553694

[B2] Aggarwal B. B. Ichikawa H. Garodia P. Weerasinghe P. Sethi G. Bhatt I. D. (2006). From traditional ayurvedic medicine to modern medicine: Identification of therapeutic targets for suppression of inflammation and cancer. Expert Opin. Ther. targets 10, 87–118. 10.1517/14728222.10.1.87 16441231

[B3] Akrami-Hasan-Kohal M. Eskandari M. Solouk A. (2021). Silk fibroin hydrogel/dexamethasone sodium phosphate loaded chitosan nanoparticles as a potential drug delivery system. Colloids surfaces. B, Biointerfaces 205, 111892. 10.1016/j.colsurfb.2021.111892 34107443

[B4] Al Alawi L. Soteriades E. S. Paulo M. S. Östlundh L. Grivna M. Al Maskari F. (2020). Environmental assessment of cytotoxic drugs in healthcare settings: Protocol for a systematic review and meta-analysis. Syst. Rev. 9, 242. 10.1186/s13643-020-01494-4 33076972 PMC7574301

[B5] Al Saqr A. Wani S. U. D. Gangadharappa H. V. Aldawsari M. F. Khafagy E. S. Lila A. S. A. (2021). Enhanced cytotoxic activity of docetaxel-loaded silk fibroin nanoparticles against breast cancer cells. Polymers 13, 1416. 10.3390/polym13091416 33925581 PMC8123888

[B6] Al-Mansoori L. Elsinga P. Goda S. K. (2021). Bio-vehicles of cytotoxic drugs for delivery to tumor specific targets for cancer precision therapy. Biomed. Pharmacother. 144, 112260. 10.1016/j.biopha.2021.112260 34607105

[B7] Altman G. H. Horan R. L. Lu H. H. Moreau J. Martin I. Richmond J. C. (2002). Silk matrix for tissue engineered anterior cruciate ligaments. Biomaterials 23, 4131–4141. 10.1016/s0142-9612(02)00156-4 12182315

[B8] Arumugam M. Murugesan B. Pandiyan N. Chinnalagu D. K. Rangasamy G. Mahalingam S. (2021). Ornamental morphology of ionic liquid functionalized ternary doped N, P, F and N, B, F-reduced graphene oxide and their prevention activities of bacterial biofilm-associated with orthopedic implantation. Mater. Sci. Eng. C, Mater. Biol. Appl. 123, 1122–1132. 10.1016/j.msec.2019.01.052 30812996

[B9] Bakos O. Lawson C. Rouleau S. Tai L. H. (2018). Combining surgery and immunotherapy: Turning an immunosuppressive effect into a therapeutic opportunity. J. Immunother. Cancer 6, 86. 10.1186/s40425-018-0398-7 30176921 PMC6122574

[B10] Belbéoch C. Lejeune J. Vroman P. Salaün F. (2021). Silkworm and spider silk electrospinning: A review. Environ. Chem. Lett. 19, 1737–1763. 10.1007/s10311-020-01147-x 33424525 PMC7779161

[B11] Bian X. Wu P. Sha H. Qian H. Wang Q. Cheng L. (2016). Anti-EGFR-iRGD recombinant protein conjugated silk fibroin nanoparticles for enhanced tumor targeting and antitumor efficiency. Onco Targets Ther. 9, 3153–3162. 10.2147/OTT.S100678 27313461 PMC4892850

[B12] Bini E. Knight D. P. Kaplan D. L. (2004). Mapping domain structures in silks from insects and spiders related to protein assembly. J. Mol. Biol. 335, 27–40. 10.1016/j.jmb.2003.10.043 14659737

[B13] Blamires S. J. Spicer P. T. Flanagan P. J. (2020). Spider silk biomimetics programs to inform the development of new wearable technologies. Front. Mater. 7. 10.3389/fmats.2020.00029

[B14] Boreel D. F. Span P. N. Heskamp S. Adema G. J. Bussink J. (2021). Targeting oxidative phosphorylation to increase the efficacy of radio- and immune-combination therapy. Clin. Cancer Res. 27, 2970–2978. 10.1158/1078-0432.CCR-20-3913 33419779

[B15] Cao W. Chen H. D. Yu Y. W. Li N. Chen W. Q. (2021). Changing profiles of cancer burden worldwide and in China: A secondary analysis of the global cancer statistics 2020. Chin. Med. J. Engl. 134, 783–791. 10.1097/CM9.0000000000001474 33734139 PMC8104205

[B16] Cao Y. Wang B. (2009). Biodegradation of silk biomaterials. Int. J. Mol. Sci. 10, 1514–1524. 10.3390/ijms10041514 19468322 PMC2680630

[B17] Chen B. Q. Kankala R. K. He G. Y. Yang D. Y. Li G. P. Wang P. (2018). Supercritical fluid-Assisted fabrication of indocyanine green-encapsulated silk fibroin nanoparticles for dual-triggered cancer therapy. ACS Biomaterials Sci. Eng. 4, 3487–3497. 10.1021/acsbiomaterials.8b00705 33450797

[B18] Chen G. Wei R. Huang X. Wang F. Chen Z. (2020). Synthesis and assessment of sodium alginate-modified silk fibroin microspheres as potential hepatic arterial embolization agent. Int. J. Biol. Macromol. 155, 1450–1459. 10.1016/j.ijbiomac.2019.11.122 31734365

[B19] Chen J. Ding J. Xu W. Sun T. Xiao H. Zhuang X. (2017). Correction to receptor and microenvironment dual-recognizable nanogel for targeted chemotherapy of highly metastatic malignancy. Nano Lett. 17, 5180. 10.1021/acs.nanolett.7b03017 28718652

[B20] Chen J. Zhan Y. Wang Y. Han D. Tao B. Luo Z. (2018b). Chitosan/silk fibroin modified nanofibrous patches with mesenchymal stem cells prevent heart remodeling post-myocardial infarction in rats. Acta Biomater. 80, 154–168. 10.1016/j.actbio.2018.09.013 30218777

[B21] Chen X. Deng L. Jiang X. Wu T. (2016). Chinese herbal medicine for oesophageal cancer. Cochrane database Syst. Rev., Cd004520. 10.1002/14651858.CD004520 26799001 PMC10217096

[B22] Chen X. Shao Z. Knight D. P. Vollrath F. (2007). Conformation transition kinetics of *Bombyx mori* silk protein. Proteins 68, 223–231. 10.1002/prot.21414 17436322

[B23] Chen Y. Wu H. Yang T. Zhou G. Chen Y. Wang J. (2021). Biomimetic nucleation of metal-organic frameworks on silk fibroin nanoparticles for designing core-shell-structured pH-responsive anticancer drug carriers. ACS Appl. Mater. Interfaces 13, 47371–47381. 10.1021/acsami.1c13405 34582680

[B24] Cheng Y. Koh L. D. Li D. Ji B. Han M. Y. Zhang Y. W. (2014). On the strength of β-sheet crystallites of *Bombyx mori* silk fibroin. J. R. Soc. Interface 11, 20140305. 10.1098/rsif.2014.0305 24789564 PMC4032545

[B25] Chirila T. V. Suzuki S. Bray L. J. Barnett N. L. Harkin D. G. (2013). Evaluation of silk sericin as a biomaterial: *In vitro* growth of human corneal limbal epithelial cells on *Bombyx mori* sericin membranes. Prog. biomaterials 2, 14. 10.1186/2194-0517-2-14 PMC5151120 29470674

[B26] Chis A. A. Arseniu A. M. Morgovan C. Dobrea C. M. Frum A. Juncan A. M. (2022). Biopolymeric prodrug systems as potential antineoplastic Therapy. Pharmaceutics 14, 1773. 10.3390/pharmaceutics14091773 36145522 PMC9505808

[B27] Choi M. Choi D. Hong J. (2018). Multilayered controlled drug release silk fibroin nanofilm by manipulating secondary structure. Biomacromolecules 19, 3096–3103. 10.1021/acs.biomac.8b00687 29894631

[B28] Cortés J. Kim S. B. Chung W. P. Im S. A. Park Y. H. Hegg R. (2022). Trastuzumab deruxtecan versus trastuzumab emtansine for breast cancer. N. Engl. J. Med. 386, 1143–1154. 10.1056/NEJMoa2115022 35320644

[B29] Crakes K. R. Herrera C. Morgan J. L. Olstad K. Hessell A. J. Ziprin P. (2020). Efficacy of silk fibroin biomaterial vehicle for *in vivo* mucosal delivery of Griffithsin and protection against HIV and SHIV infection *ex vivo* . J. Int. AIDS Soc. 23, e25628. 10.1002/jia2.25628 33073530 PMC7569169

[B30] Crivelli B. Bari E. Perteghella S. Catenacci L. Sorrenti M. Mocchi M. (2019). Silk fibroin nanoparticles for celecoxib and curcumin delivery: ROS-scavenging and anti-inflammatory activities in an *in vitro* model of osteoarthritis. Eur. J. Pharm. Biopharm. 137, 37–45. 10.1016/j.ejpb.2019.02.008 30772432

[B31] Desai S. K. Mondal D. Bera S. (2021). Polyurethane-functionalized starch nanocrystals as anti-tuberculosis drug carrier. Sci. Rep. 11, 8331. 10.1038/s41598-021-86767-1 33859215 PMC8050055

[B32] Deshpande R. Shukla S. Kale A. Deshmukh N. Nisal A. Venugopalan P. (2022). Silk fibroin microparticle scaffold for use in bone void filling: Safety and efficacy studies. ACS Biomaterials Sci. Eng. 8, 1226–1238. 10.1021/acsbiomaterials.1c01103 35166518

[B33] Diez-Echave P. Ruiz-Malagón A. J. Molina-Tijeras J. A. Hidalgo-García L. Vezza T. Cenis-Cifuentes L. (2021). Silk fibroin nanoparticles enhance quercetin immunomodulatory properties in DSS-induced mouse colitis. Int. J. Pharm. 606, 120935. 10.1016/j.ijpharm.2021.120935 34310954

[B34] Ding B. Wahid M. A. Wang Z. Xie C. Thakkar A. Prabhu S. (2017). Triptolide and celastrol loaded silk fibroin nanoparticles show synergistic effect against human pancreatic cancer cells. Nanoscale 9, 11739–11753. 10.1039/c7nr03016a 28782773 PMC5648537

[B35] Eivazzadeh-Keihan R. Khalili F. Aliabadi H. A. M. Maleki A. Madanchi H. Ziabari E. Z. (2020). Alginate hydrogel-polyvinyl alcohol/silk fibroin/magnesium hydroxide nanorods: A novel scaffold with biological and antibacterial activity and improved mechanical properties. Int. J. Biol. Macromol. 162, 1959–1971. 10.1016/j.ijbiomac.2020.08.090 32814101

[B36] El Fawal G. Abu-Serie M. M. Mo X. Wang H. (2021). Diethyldithiocarbamate/silk fibroin/polyethylene oxide nanofibrous for cancer therapy: Fabrication, characterization and *in vitro* evaluation. Int. J. Biol. Macromol. 193, 293–299. 10.1016/j.ijbiomac.2021.10.039 34656539

[B37] Estey T. Kang J. Schwendeman S. P. Carpenter J. F. (2006). BSA degradation under acidic conditions: A model for protein instability during release from PLGA delivery systems. J. Pharm. Sci. 95, 1626–1639. 10.1002/jps.20625 16729268

[B38] Farokhi M. Mottaghitalab F. Reis R. L. Ramakrishna S. Kundu S. C. (2020). Functionalized silk fibroin nanofibers as drug carriers: Advantages and challenges. J. Control. release official J. Control. Release Soc. 321, 324–347. 10.1016/j.jconrel.2020.02.022 32061791

[B39] Fernández-García L. Marí-Buyé N. Barios J. A. Madurga R. Elices M. Pérez-Rigueiro J. (2016). Safety and tolerability of silk fibroin hydrogels implanted into the mouse brain. Acta Biomater. 45, 262–275. 10.1016/j.actbio.2016.09.003 27592819

[B40] Florczak A. Deptuch T. Kucharczyk K. Dams-Kozlowska H. (2021). Systemic and local silk-based drug delivery systems for cancer therapy. Cancers 13, 5389. 10.3390/cancers13215389 34771557 PMC8582423

[B41] Florczak A. Grzechowiak I. Deptuch T. Kucharczyk K. Kaminska A. Dams-Kozlowska H. (2020). Silk particles as carriers of therapeutic molecules for cancer treatment. Mater. (Basel) 13, 4946. 10.3390/ma13214946 PMC7663281 33158060

[B42] Florczak A. Mackiewicz A. Dams-Kozlowska H. (2014). Functionalized spider silk spheres as drug carriers for targeted cancer therapy. Biomacromolecules 15, 2971–2981. 10.1021/bm500591p 24963985

[B43] Floren M. Migliaresi C. Motta A. (2016). Processing techniques and applications of silk hydrogels in bioengineering. J. Funct. biomaterials 7, 26. 10.3390/jfb7030026 PMC5040999 27649251

[B44] Fuster M. G. Carissimi G. Montalbán M. G. Víllora G. (2021). Antitumor activity of rosmarinic acid-loaded silk fibroin nanoparticles on HeLa and MCF-7 Cells. Polymers 13, 3169. 10.3390/polym13183169 34578069 PMC8467615

[B45] Gangrade A. Mandal B. B. (2020). Drug delivery of anticancer drugs from injectable 3D porous silk scaffold for prevention of gastric cancer growth and recurrence. ACS Biomaterials Sci. Eng. 6, 6195–6206. 10.1021/acsbiomaterials.0c01043 33449660

[B46] Gao D. Lo P. C. (2018). Polymeric micelles encapsulating pH-responsive doxorubicin prodrug and glutathione-activated zinc(II) phthalocyanine for combined chemotherapy and photodynamic therapy. J. Control. release official J. Control. Release Soc. 282, 46–61. 10.1016/j.jconrel.2018.04.030 29673646

[B47] Gao Y. Xie J. Chen H. Gu S. Zhao R. Shao J. (2014). Nanotechnology-based intelligent drug design for cancer metastasis treatment. Biotechnol. Adv. 32, 761–777. 10.1016/j.biotechadv.2013.10.013 24211475

[B48] Gholipourmalekabadi M. Sapru S. Samadikuchaksaraei A. Reis R. L. Kaplan D. L. Kundu S. C. (2020). Silk fibroin for skin injury repair: Where do things stand? Adv. drug Deliv. Rev. 153, 28–53. 10.1016/j.addr.2019.09.003 31678360

[B49] Gosline J. M. Guerette P. A. Ortlepp C. S. Savage K. N. (1999). The mechanical design of spider silks: From fibroin sequence to mechanical function. J. Exp. Biol. 202, 3295–3303. 10.1242/jeb.202.23.3295 10562512

[B50] Gou S. Huang Y. Sung J. Xiao B. Merlin D. (2019a). Silk fibroin-based nanotherapeutics: Application in the treatment of colonic diseases. Nanomedicine (Lond) 14, 2373–2378. 10.2217/nnm-2019-0058 31290366 PMC7026768

[B51] Gou S. Huang Y. Wan Y. Ma Y. Zhou X. Tong X. (2019b). Multi-bioresponsive silk fibroin-based nanoparticles with on-demand cytoplasmic drug release capacity for CD44-targeted alleviation of ulcerative colitis. Biomaterials 212, 39–54. 10.1016/j.biomaterials.2019.05.012 31103945

[B52] Gregory D. A. Zhang Y. Smith P. J. Zhao X. Ebbens S. J. (2016). Reactive inkjet printing of biocompatible enzyme powered silk micro-rockets. Small 12, 4048–4055. 10.1002/smll.201600921 27345008

[B53] Guo X. Lin N. Lu S. Zhang F. Zuo B. (2021). Preparation and biocompatibility characterization of silk fibroin 3D scaffolds. ACS Appl. Bio Mater. 4, 1369–1380. 10.1021/acsabm.0c01239 35014488

[B54] Gupta V. Aseh A. Ríos C. N. Aggarwal B. B. Mathur A. B. (2009). Fabrication and characterization of silk fibroin-derived curcumin nanoparticles for cancer therapy. Int. J. nanomedicine 4, 115–122. 10.2147/ijn.s5581 19516890 PMC2720745

[B55] Hassani Besheli N. Mottaghitalab F. Eslami M. Gholami M. Kundu S. C. Kaplan D. L. (2017). Sustainable release of vancomycin from silk fibroin nanoparticles for treating severe bone infection in rat tibia osteomyelitis model. ACS Appl. Mater. Interfaces 9, 5128–5138. 10.1021/acsami.6b14912 28106379

[B56] Hassanzadeh P. Arbabi E. Rostami F. (2021). Coating of ferulic acid-loaded silk fibroin nanoparticles with neutrophil membranes: A promising strategy against the acute pancreatitis. Life Sci. 270, 119128. 10.1016/j.lfs.2021.119128 33508299

[B57] He J. Fan K. Yan X. (2019). Ferritin drug carrier (FDC) for tumor targeting therapy. J. Control. release official J. Control. Release Soc. 311-312, 288–300. 10.1016/j.jconrel.2019.09.002 31494184

[B58] Hitchcock J. White A. L. Hondow N. Hughes T. A. Dupont H. Biggs S. (2020). Metal-shell nanocapsules for the delivery of cancer drugs. J. colloid interface Sci. 567, 171–180. 10.1016/j.jcis.2019.12.018 32045739

[B59] Hofer M. Winter G. Myschik J. (2012). Recombinant spider silk particles for controlled delivery of protein drugs. Biomaterials 33, 1554–1562. 10.1016/j.biomaterials.2011.10.053 22079006

[B60] Holland C. Numata K. Rnjak-Kovacina J. Seib F. P. (2019). The biomedical use of silk: Past, present, future. Adv. Healthc. Mater. 8, e1800465. 10.1002/adhm.201800465 30238637

[B61] Hong H. Seo Y. B. Kim D. Y. Lee J. S. Lee Y. J. Lee H. (2020a). Digital light processing 3D printed silk fibroin hydrogel for cartilage tissue engineering. Biomaterials 232, 119679. 10.1016/j.biomaterials.2019.119679 31865191

[B62] Hong S. Choi D. W. Kim H. N. Park C. G. Lee W. Park H. H. (2020b). Protein-based nanoparticles as drug delivery systems. Pharmaceutics 12, 604. 10.3390/pharmaceutics12070604 32610448 PMC7407889

[B63] Hu J. Wang M. Xiao X. Zhang B. Xie Q. Xu X. (2020a). A novel long-acting azathioprine polyhydroxyalkanoate nanoparticle enhances treatment efficacy for systemic lupus erythematosus with reduced side effects. Nanoscale 12, 10799–10808. 10.1039/d0nr01308k 32391836

[B64] Hu L. Han Y. Ling S. Huang Y. Yao J. Shao Z. (2020b). Direct Observation of Native Silk fibroin conformation in silk gland of bombyx mori silkworm. ACS Biomaterials Sci. Eng. 6, 1874–1879. 10.1021/acsbiomaterials.9b01586 33455357

[B65] Hudiță A. Radu I. C. Zaharia C. Ion A. C. Ginghină O. Gălățeanu B. (2021). Bio- and hemo-compatible silk fibroin PEGylated nanocarriers for 5-fluorouracil chemotherapy in colorectal cancer: *In vitro* studies. Pharmaceutics 13, 755. 10.3390/pharmaceutics13050755 34069731 PMC8160811

[B66] Huot A. Lefèvre T. Rioux-Dubé J. F. Paquet-Mercier F. Nault A. P. Auger M. (2015). Effect of mechanical deformation on the structure of regenerated *Bombyx mori* silk fibroin films as revealed using Raman and infrared spectroscopy. Appl. Spectrosc. 69, 689–698. 10.1366/14-07776 25954973

[B67] Jiang H. Li M. Du K. Ma C. Cheng Y. Wang S. (2021). Traditional Chinese medicine for adjuvant treatment of breast cancer: Taohong siwu decoction. Chin. Med. 16, 129. 10.1186/s13020-021-00539-7 34857023 PMC8638166

[B68] Kang E. J. Baek Y. M. Hahm E. Lee S. H. Pham X. H. Noh M. S. (2019). Functionalized β-cyclodextrin immobilized on Ag-embedded silica nanoparticles as a drug carrier. Int. J. Mol. Sci. 20, 315. 10.3390/ijms20020315 30646562 PMC6359520

[B69] Kapoor S. Kundu S. C. (2016). Silk protein-based hydrogels: Promising advanced materials for biomedical applications. Acta Biomater. 31, 17–32. 10.1016/j.actbio.2015.11.034 26602821

[B70] Kaushik I. Ramachandran S. Srivastava S. K. (2019). CRISPR-Cas9: A multifaceted therapeutic strategy for cancer treatment. Semin. Cell Dev. Biol. 96, 4–12. 10.1016/j.semcdb.2019.04.018 31054324 PMC6829064

[B71] Khosravimelal S. Chizari M. Farhadihosseinabadi B. Moosazadeh M. M. Gholipourmalekabadi M. (2021). Fabrication and characterization of an antibacterial chitosan/silk fibroin electrospun nanofiber loaded with a cationic peptide for wound-dressing application. J. Mater Sci. Mater Med. 32, 114. 10.1007/s10856-021-06542-6 34455501 PMC8403119

[B72] Kim J. H. Sheikh F. A. Ju H. W. Park H. J. Moon B. M. Lee O. J. (2014). 3D silk fibroin scaffold incorporating titanium dioxide (TiO2) nanoparticle (NPs) for tissue engineering. Int. J. Biol. Macromol. 68, 158–168. 10.1016/j.ijbiomac.2014.04.045 24794196

[B73] Kim S. H. Hong H. Ajiteru O. Sultan M. T. Lee Y. J. Lee J. S. (2021). 3D bioprinted silk fibroin hydrogels for tissue engineering. Nat. Protoc. 16, 5484–5532. 10.1038/s41596-021-00622-1 34716451

[B74] Kim S. Y. Naskar D. Kundu S. C. Bishop D. P. Doble P. A. Boddy A. V. (2015). Formulation of biologically-inspired silk-based drug carriers for pulmonary delivery targeted for lung cancer. Sci. Rep. 5, 11878. 10.1038/srep11878 26234773 PMC4522685

[B75] Koh L.-D. Cheng Y. Teng C.-P. Khin Y.-W. Loh X.-J. Tee S.-Y. (2015). Structures, mechanical properties and applications of silk fibroin materials. Prog. Polym. Sci. 46, 86–110. 10.1016/j.progpolymsci.2015.02.001

[B76] Kunde S. S. Wairkar S. (2022). Targeted delivery of albumin nanoparticles for breast cancer: A review. Colloids surfaces. B, Biointerfaces 213, 112422. 10.1016/j.colsurfb.2022.112422 35231688

[B77] Lammel A. S. Hu X. Park S. H. Kaplan D. L. Scheibel T. R. (2010). Controlling silk fibroin particle features for drug delivery. Biomaterials 31, 4583–4591. 10.1016/j.biomaterials.2010.02.024 20219241 PMC2846964

[B78] Lazaris A. Arcidiacono S. Huang Y. Zhou J. F. Duguay F. Chretien N. (2002). Spider silk fibers spun from soluble recombinant silk produced in mammalian cells. Science 295, 472–476. 10.1126/science.1065780 11799236

[B79] Lee O. J. Lee J. M. Kim J. H. Kim J. Kweon H. Jo Y. Y. (2012). Biodegradation behavior of silk fibroin membranes in repairing tympanic membrane perforations. J. Biomed. Mater. Res. Part A 100, 2018–2026. 10.1002/jbm.a.33308 22581612

[B80] Lehmann T. Vaughn A. E. Seal S. Liechty K. W. Zgheib C. (2022). Silk fibroin-based therapeutics for impaired wound healing. Pharmaceutics 14, 651. 10.3390/pharmaceutics14030651 35336024 PMC8949428

[B81] Li H. Tian J. Wu A. Wang J. Ge C. Sun Z. (2016). Self-assembled silk fibroin nanoparticles loaded with binary drugs in the treatment of breast carcinoma. Int. J. nanomedicine 11, 4373–4380. 10.2147/IJN.S108633 27621628 PMC5015876

[B82] Li L. Puhl S. Meinel L. Germershaus O. (2014). Silk fibroin layer-by-layer microcapsules for localized gene delivery. Biomaterials 35, 7929–7939. 10.1016/j.biomaterials.2014.05.062 24930849

[B83] Li W. Cao Z. Liu R. Liu L. Li H. Li X. (2019). AuNPs as an important inorganic nanoparticle applied in drug carrier systems. Artif. Cells Nanomed Biotechnol. 47, 4222–4233. 10.1080/21691401.2019.1687501 31713452

[B84] Li Y. Yang Y. Yang L. Zeng Y. Gao X. Xu H. (2017). Poly(ethylene glycol)-modified silk fibroin membrane as a carrier for limbal epithelial stem cell transplantation in a rabbit LSCD model. Stem Cell Res. Ther. 8, 256. 10.1186/s13287-017-0707-y 29116027 PMC5678789

[B85] Liebsch C. Bucan V. Menger B. Köhne F. Waldmann K. H. Vaslaitis D. (2018). Preliminary investigations of spider silk in wounds *in vivo* - implications for an innovative wound dressing. Burns 44, 1829–1838. 10.1016/j.burns.2018.03.016 30057335

[B86] Lin L. Zhou Y. Quan G. Pan X. Wu C. (2021). The rough inhalable ciprofloxacin hydrochloride microparticles based on silk fibroin for non-cystic fibrosis bronchiectasis therapy with good biocompatibility. Int. J. Pharm. 607, 120974. 10.1016/j.ijpharm.2021.120974 34358540

[B87] Liu H. Li X. Zhou G. Fan H. Fan Y. (2011). Electrospun sulfated silk fibroin nanofibrous scaffolds for vascular tissue engineering. Biomaterials 32, 3784–3793. 10.1016/j.biomaterials.2011.02.002 21376391

[B88] Liu Y. Huang W. Meng M. Chen M. Cao C. (2021). Progress in the application of spider silk protein in medicine. J. biomaterials Appl. 36, 859–871. 10.1177/08853282211003850 33853426

[B89] Lu Q. Zhang B. Li M. Zuo B. Kaplan D. L. Huang Y. (2011). Degradation mechanism and control of silk fibroin. Biomacromolecules 12, 1080–1086. 10.1021/bm101422j 21361368 PMC3404841

[B90] Luetchford K. A. Chaudhuri J. B. De Bank P. A. (2020). Silk fibroin/gelatin microcarriers as scaffolds for bone tissue engineering. Mater. Sci. Eng. C, Mater. Biol. Appl. 106, 110116. 10.1016/j.msec.2019.110116 31753329 PMC6891254

[B91] Luo Q. Zhang L. Luo C. Jiang M. (2019). Emerging strategies in cancer therapy combining chemotherapy with immunotherapy. Cancer Lett. 454, 191–203. 10.1016/j.canlet.2019.04.017 30998963

[B92] Lv Z. D. Song H. M. Niu Z. H. Nie G. Zheng S. Xu Y. Y. (2021). Efficacy and safety of albumin-bound paclitaxel compared to docetaxel as neoadjuvant chemotherapy for HER2-negative breast cancer. Front. Oncol. 11, 760655. 10.3389/fonc.2021.760655 35087749 PMC8787090

[B93] Ma Y. Canup B. S. B. Tong X. Dai F. Xiao B. (2020). Multi-responsive silk fibroin-based nanoparticles for drug delivery. Front. Chem. 8, 585077. 10.3389/fchem.2020.585077 33240846 PMC7670059

[B94] Mancipe Castro L. M. García A. J. Guldberg R. E. (2021). Biomaterial strategies for improved intra-articular drug delivery. J. Biomed. Mater. Res. Part A 109, 426–436. 10.1002/jbm.a.37074 PMC8906235 32780515

[B95] Mao B. Liu C. Zheng W. Li X. Ge R. Shen H. (2018). Cyclic cRGDfk peptide and chlorin e6 functionalized silk fibroin nanoparticles for targeted drug delivery and photodynamic therapy. Biomaterials 161, 306–320. 10.1016/j.biomaterials.2018.01.045 29427926

[B96] Mathur A. B. Gupta V. (2010). Silk fibroin-derived nanoparticles for biomedical applications. Nanomedicine (Lond) 5, 807–820. 10.2217/nnm.10.51 20662650

[B97] Mazzaferro S. Bouchemal K. Ponchel G. (2013). Oral delivery of anticancer drugs III: Formulation using drug delivery systems. Drug Discov. today 18, 99–104. 10.1016/j.drudis.2012.08.007 22981667

[B98] Mehreen E. Shaukat A. Hafiz M. T. Rabia M. Muhammad F. B. (2021). Sericin and fibroin nanoparticles—natural product for cancer therapy: A comprehensive review. Int. J. Polym. Mater. Polym. Biomaterials 70, 256–269. 10.1080/00914037.2019.1706515

[B99] Melke J. Midha S. Ghosh S. Ito K. Hofmann S. (2016). Silk fibroin as biomaterial for bone tissue engineering. Acta Biomater. 31, 1–16. 10.1016/j.actbio.2015.09.005 26360593

[B100] Messina C. Merz V. Frisinghelli M. Trentin C. Grego E. Veccia A. (2019). Adjuvant chemotherapy in resected bile duct cancer: A systematic review and meta-analysis of randomized trials. Crit. Rev. Oncol. Hematol. 143, 124–129. 10.1016/j.critrevonc.2019.09.002 31563828

[B101] Ming J. Pan F. Zuo B. (2015). Influence factors analysis on the formation of silk I structure. Int. J. Biol. Macromol. 75, 398–401. 10.1016/j.ijbiomac.2015.02.002 25677178

[B102] Minoura N. Aiba S. Higuchi M. Gotoh Y. Tsukada M. Imai Y. (1995). Attachment and growth of fibroblast cells on silk fibroin. Biochem. biophysical Res. Commun. 208, 511–516. 10.1006/bbrc.1995.1368 7695601

[B103] Mohammed M. A. Syeda J. T. M. Wasan K. M. Wasan E. K. (2017). An Overview of chitosan nanoparticles and its application in non-parenteral drug delivery. Pharmaceutics 9, 53. 10.3390/pharmaceutics9040053 29156634 PMC5750659

[B104] Moin A. Wani S. U. D. Osmani R. A. Abu Lila A. S. Khafagy E. S. Arab H. H. (2021). Formulation, characterization, and cellular toxicity assessment of tamoxifen-loaded silk fibroin nanoparticles in breast cancer. Drug Deliv. 28, 1626–1636. 10.1080/10717544.2021.1958106 34328806 PMC8330732

[B105] Montalbán M. G. Coburn J. M. Lozano-Pérez A. A. Cenis J. L. Víllora G. Kaplan D. L. (2018). Production of curcumin-loaded silk fibroin nanoparticles for cancer therapy. Nanomater. (Basel) 8, 126. 10.3390/nano8020126 PMC5853757 29495296

[B106] Mottaghitalab F. Farokhi M. Shokrgozar M. A. Atyabi F. Hosseinkhani H. (2015). Silk fibroin nanoparticle as a novel drug delivery system. J. Control. release official J. Control. Release Soc. 206, 161–176. 10.1016/j.jconrel.2015.03.020 25797561

[B107] Mottaghitalab F. Kiani M. Farokhi M. Kundu S. C. Reis R. L. Gholami M. (2017). Targeted delivery system based on gemcitabine-loaded silk fibroin nanoparticles for lung cancer therapy. ACS Appl. Mater. Interfaces 9, 31600–31611. 10.1021/acsami.7b10408 28836425

[B108] Myung S. J. Kim H. S. Kim Y. (2020). Fluorescent silk fibroin nanoparticles prepared using a reverse microemulsion. Macromol. Res. 16 (7), 604–608. 10.1007/bf03218567

[B109] Nazarov R. Jin H. J. Kaplan D. L. (2004). Porous 3-D scaffolds from regenerated silk fibroin. Biomacromolecules 5, 718–726. 10.1021/bm034327e 15132652

[B110] Newell H. Sausville E. (2016). Cytotoxic drugs: Past, present and future. Cancer Chemother. Pharmacol. 77, 1. 10.1007/s00280-015-2917-2 26703129

[B111] Nuvola G. Rizzo A. Mollica V. Massari F. (2022). The dilemma of neoadjuvant and adjuvant therapy in urothelial carcinoma: Will immunotherapy solve the problem? Immunotherapy 14, 171–174. 10.2217/imt-2021-0324 35000464

[B112] Oliveira I. M. Gonçalves C. Shin M. E. Lee S. Reis R. L. Khang G. (2020). Anti-inflammatory properties of injectable betamethasone-loaded tyramine-modified gellan gum/silk fibroin hydrogels. Biomolecules 10, 1456. 10.3390/biom10101456 33080875 PMC7603075

[B113] Pandey V. Haider T. Chandak A. R. Chakraborty A. Banerjee S. Soni V. (2020). Surface modified silk fibroin nanoparticles for improved delivery of doxorubicin: Development, characterization, *in-vitro* studies. Int. J. Biol. Macromol. 164, 2018–2027. 10.1016/j.ijbiomac.2020.07.326 32758604

[B114] Pascoli M. de Lima R. Fraceto L. F. (2018). Zein nanoparticles and strategies to improve colloidal stability: A mini-review. Front. Chem. 6, 6. 10.3389/fchem.2018.00006 29473032 PMC5810256

[B115] Patwa R. Soundararajan N. Mulchandani N. Bhasney S. M. Shah M. Kumar S. (2018). Silk nano-discs: A natural material for cancer therapy. Biopolymers 109, e23231. 10.1002/bip.23231 30515775

[B116] Pham D. T. Saelim N. Tiyaboonchai W. (2019). Alpha mangostin loaded crosslinked silk fibroin-based nanoparticles for cancer chemotherapy. Colloids surfaces. B, Biointerfaces 181, 705–713. 10.1016/j.colsurfb.2019.06.011 31228853

[B117] Pham D. T. Tiyaboonchai W. (2020). Fibroin nanoparticles: A promising drug delivery system. Drug Deliv. 27, 431–448. 10.1080/10717544.2020.1736208 32157919 PMC7144220

[B118] Qi Y. Wang H. Wei K. Yang Y. Zheng R. Y. Kim I. S. (2017). A review of structure construction of silk fibroin biomaterials from single structures to multi-level structures. Int. J. Mol. Sci. 18, 237. 10.3390/ijms18030237 28273799 PMC5372488

[B119] Qi Z. Cao J. Tao X. Wu X. Kundu S. C. Lu S. (2021). Silk fibroin microneedle patches for the treatment of insomnia. Pharmaceutics 13, 2198. 10.3390/pharmaceutics13122198 34959479 PMC8704547

[B120] Qian K. Y. Song Y. Yan X. Dong L. Xue J. Xu Y. (2020). Injectable ferrimagnetic silk fibroin hydrogel for magnetic hyperthermia ablation of deep tumor. Biomaterials 259, 120299. 10.1016/j.biomaterials.2020.120299 32827797

[B121] Qu J. Liu Y. Yu Y. Li J. Luo J. Li M. (2014). Silk fibroin nanoparticles prepared by electrospray as controlled release carriers of cisplatin. Mater. Sci. Eng. C, Mater. Biol. Appl. 44, 166–174. 10.1016/j.msec.2014.08.034 25280693

[B122] Qu J. Wang W. Feng Y. Niu L. Li M. Yang J. (2019). Cationic antheraea pernyi silk fibroin-modified adenovirus-mediated ING4 and IL-24 dual gene coexpression vector suppresses the growth of hepatoma carcinoma cells. Int. J. nanomedicine 14, 9745–9761. 10.2147/IJN.S230693 31849466 PMC6911339

[B123] Rajkhowa R. Wang L. Wang X. (2008). Ultra-fine silk powder preparation through rotary and ball milling. Powder Technol. 185, 87–95. 10.1016/j.powtec.2008.01.005

[B124] Rising A. Johansson J. (2015). Toward spinning artificial spider silk. Nat. Chem. Biol. 11, 309–315. 10.1038/nchembio.1789 25885958

[B125] Rodriguez-Nogales A. Algieri F. De Matteis L. Lozano-Perez A. A. Garrido-Mesa J. Vezza T. (2016). Intestinal anti-inflammatory effects of RGD-functionalized silk fibroin nanoparticles in trinitrobenzenesulfonic acid-induced experimental colitis in rats. Int. J. nanomedicine 11, 5945–5958. 10.2147/IJN.S116479 27877040 PMC5108622

[B126] Rusa C. C. Bridges C. Ha S.-W. Tonelli A. E. (2005). Conformational changes induced in *Bombyx mori* silk fibroin by cyclodextrin inclusion complexation. Macromolecules 38, 5640–5646. 10.1021/ma050340a

[B127] Salehi S. Koeck K. Scheibel T. (2020). Spider silk for tissue engineering applications. Molecules 25, 737. 10.3390/molecules25030737 32046280 PMC7037138

[B128] Scheller J. Gührs K. H. Grosse F. Conrad U. (2001). Production of spider silk proteins in tobacco and potato. Nat. Biotechnol. 19, 573–577. 10.1038/89335 11385464

[B129] Sharafat-Vaziri A. Khorasani S. Darzi M. Saffarian Z. Alizadeh Z. Tahmasebi M. N. (2020). Safety and efficacy of engineered tissue composed of silk fibroin/collagen and autologous chondrocytes in two patients with cartilage defects: A pilot clinical trial study. Knee 27, 1300–1309. 10.1016/j.knee.2020.06.015 33010742

[B130] Shen X. Zhang Y. Gu Y. Xu Y. Liu Y. Li B. (2016). Sequential and sustained release of SDF-1 and BMP-2 from silk fibroin-nanohydroxyapatite scaffold for the enhancement of bone regeneration. Biomaterials 106, 205–216. 10.1016/j.biomaterials.2016.08.023 27566869

[B131] Song D. W. Kim S. H. Kim H. H. Lee K. H. Ki C. S. Park Y. H. (2016). Multi-biofunction of antimicrobial peptide-immobilized silk fibroin nanofiber membrane: Implications for wound healing. Acta Biomater. 39, 146–155. 10.1016/j.actbio.2016.05.008 27163404

[B132] Song J. E. Tripathy N. Lee D. H. Park J. H. Khang G. (2018). Quercetin inlaid silk fibroin/hydroxyapatite scaffold promotes enhanced osteogenesis. ACS Appl. Mater. Interfaces 10, 32955–32964. 10.1021/acsami.8b08119 30188112

[B133] Spiliopoulou P. Hinsley S. McNeish I. A. Roxburgh P. Glasspool R. (2021). Metronomic oral cyclophosphamide in relapsed ovarian cancer. Int. J. Gynecol. Cancer 31, 1037–1044. 10.1136/ijgc-2021-002467 34016703

[B134] Stinson J. A. Palmer C. R. Miller D. P. Li A. B. Lightner K. Jost H. (2020). Thin silk fibroin films as a dried format for temperature stabilization of inactivated polio vaccine. Vaccine 38, 1652–1660. 10.1016/j.vaccine.2019.12.062 31959422 PMC7176408

[B135] Subia B. Chandra S. Talukdar S. Kundu S. C. (2014). Folate conjugated silk fibroin nanocarriers for targeted drug delivery. Integr. Biol. (Camb) 6, 203–214. 10.1039/c3ib40184g 24345855

[B136] Subia B. Dey T. Sharma S. Kundu S. C. (2015). Target specific delivery of anticancer drug in silk fibroin based 3D distribution model of bone-breast cancer cells. ACS Appl. Mater. Interfaces 7, 2269–2279. 10.1021/am506094c 25557227

[B137] Sun W. Gregory D. A. Tomeh M. A. Zhao X. (2021). Silk fibroin as a functional biomaterial for tissue engineering. Int. J. Mol. Sci. 22, 1499. 10.3390/ijms22031499 33540895 PMC7867316

[B138] Sung H. Ferlay J. Siegel R. Laversanne M. Soerjomataram I. Jemal A. (2021). Global cancer statistics 2020: GLOBOCAN estimates of incidence and mortality worldwide for 36 cancers in 185 countries. CA a cancer J. Clin. 71, 209–249. 10.3322/caac.21660 33538338

[B139] Suyamud C. Phetdee C. Jaimalai T. Prangkio P. (2021). Silk fibroin-coated liposomes as biomimetic nanocarrier for long-term release delivery system in cancer therapy. Molecules 26, 4936. 10.3390/molecules26164936 34443524 PMC8398433

[B140] Tanaka K. Inoue S. Mizuno S. (1999). Hydrophobic interaction of P25, containing Asn-linked oligosaccharide chains, with the H-L complex of silk fibroin produced by *Bombyx mori* . Insect Biochem. Mol. Biol. 29, 269–276. 10.1016/s0965-1748(98)00135-0 10319440

[B141] Tian Y. Jiang X. Chen X. Shao Z. Yang W. (2014). Doxorubicin-loaded magnetic silk fibroin nanoparticles for targeted therapy of multidrug-resistant cancer. Adv. Mater. 26, 7393–7398. 10.1002/adma.201403562 25238148

[B142] Tulay P. Galam N. Adali T. (2018). The wonders of silk fibroin biomaterials in the treatment of breast cancer. Crit. Rev. Eukaryot. gene Expr. 28, 129–134. 10.1615/CritRevEukaryotGeneExpr.2018021331 30055539

[B143] Umuhoza D. Yang F. Long D. Hao Z. Dai J. Zhao A. (2020). Strategies for tuning the biodegradation of silk fibroin-based materials for tissue engineering applications. ACS Biomaterials Sci. Eng. 6, 1290–1310. 10.1021/acsbiomaterials.9b01781 33455402

[B144] Valluzzi R. He S. J. Gido S. P. Kaplan D. (1999). *Bombyx mori* silk fibroin liquid crystallinity and crystallization at aqueous fibroin-organic solvent interfaces. Int. J. Biol. Macromol. 24, 227–236. 10.1016/s0141-8130(99)00005-7 10342769

[B145] Wang C. Fang H. Qi X. Hang C. Sun Y. Peng Z. (2019a). Silk fibroin film-coated MgZnCa alloy with enhanced *in vitro* and *in vivo* performance prepared using surface activation. Acta Biomater. 91, 99–111. 10.1016/j.actbio.2019.04.048 31028907

[B146] Wang D. Steffi C. Wang Z. Kong C. H. Lim P. N. Shi Z. (2018). Beta-cyclodextrin modified mesoporous bioactive glass nanoparticles/silk fibroin hybrid nanofibers as an implantable estradiol delivery system for the potential treatment of osteoporosis. Nanoscale 10, 18341–18353. 10.1039/c8nr05268a 30255905

[B147] Wang M. S. Du Y. B. Huang H. M. Zhu Z. L. Du S. S. Chen S. Y. (2019b). Silk fibroin peptide suppresses proliferation and induces apoptosis and cell cycle arrest in human lung cancer cells. Acta Pharmacol. Sin. 40, 522–529. 10.1038/s41401-018-0048-0 29921888 PMC6461792

[B148] Wang W. Yu Y. Jiang Y. Qu J. Niu L. Yang J. (2019c). Silk fibroin scaffolds loaded with angiogenic genes in adenovirus vectors for tissue regeneration. J. tissue Eng. Regen. Med. 13, 715–728. 10.1002/term.2819 30770653

[B149] Wang X. Yucel T. Lu Q. Hu X. Kaplan D. L. (2010). Silk nanospheres and microspheres from silk/pva blend films for drug delivery. Biomaterials 31, 1025–1035. 10.1016/j.biomaterials.2009.11.002 19945157 PMC2832579

[B150] Wang Y. Rudym D. D. Walsh A. Abrahamsen L. Kim H. J. Kim H. S. (2008). *In vivo* degradation of three-dimensional silk fibroin scaffolds. Biomaterials 29, 3415–3428. 10.1016/j.biomaterials.2008.05.002 18502501 PMC3206261

[B151] Wani S. U. D. Veerabhadrappa G. H. (2018). Silk fibroin based drug delivery applications: Promises and challenges. Curr. Drug Targets 19, 1177–1190. 10.2174/1389450119666171227205525 29283071

[B152] Wenk E. Wandrey A. J. Merkle H. P. Meinel L. (2008). Silk fibroin spheres as a platform for controlled drug delivery. J. Control. release official J. Control. Release Soc. 132, 26–34. 10.1016/j.jconrel.2008.08.005 18761384

[B153] Wu H. C. Quan D. N. Tsao C. Y. Liu Y. Terrell J. L. Luo X. (2017). Conferring biological activity to native spider silk: A biofunctionalized protein-based microfiber. Biotechnol. Bioeng. 114, 83–95. 10.1002/bit.26065 27478042

[B154] Wu N. Yu H. Sun M. Li Z. Zhao F. Ao Y. (2020). Investigation on the structure and mechanical properties of highly tunable elastomeric silk fibroin hydrogels cross-linked by γ-ray radiation. ACS Appl. Bio Mater. 3, 721–734. 10.1021/acsabm.9b01062 35019416

[B155] Wu P. Liu Q. Li R. Wang J. Zhen X. Yue G. (2013). Facile preparation of paclitaxel loaded silk fibroin nanoparticles for enhanced antitumor efficacy by locoregional drug delivery. ACS Appl. Mater. Interfaces 5, 12638–12645. 10.1021/am403992b 24274601

[B156] Wu P. Liu Q. Wang Q. Qian H. Yu L. Liu B. (2018). Novel silk fibroin nanoparticles incorporated silk fibroin hydrogel for inhibition of cancer stem cells and tumor growth. Int. J. Nanomedicine 13, 5405–5418. 10.2147/IJN.S166104 30271137 PMC6149978

[B157] Xia X. X. Xu Q. Hu X. Qin G. Kaplan D. L. (2011). Tunable self-assembly of genetically engineered silk-elastin-like protein polymers. Biomacromolecules 12, 3844–3850. 10.1021/bm201165h 21955178 PMC3224811

[B158] Xie M. Fan D. Chen Y. Zhao Z. He X. Li G. (2016). An implantable and controlled drug-release silk fibroin nanofibrous matrix to advance the treatment of solid tumour cancers. Biomaterials 103, 33–43. 10.1016/j.biomaterials.2016.06.049 27376557

[B159] Xie M. Fan D. Li Y. He X. Chen X. Chen Y. (2017). Supercritical carbon dioxide-developed silk fibroin nanoplatform for smart colon cancer therapy. Int. J. nanomedicine 12, 7751–7761. 10.2147/IJN.S145012 29118580 PMC5659230

[B160] Xin X. Wu J. Zheng A. Jiao D. Liu Y. Cao L. (2019). Delivery vehicle of muscle-derived irisin based on silk/calcium silicate/sodium alginate composite scaffold for bone regeneration. Int. J. nanomedicine 14, 1451–1467. 10.2147/IJN.S193544 30863071 PMC6390863

[B161] Xu H. L. Chen P. P. ZhuGe D. L. Zhu Q. Y. Jin B. H. Shen B. X. (2017). Liposomes with silk fibroin hydrogel core to stabilize bFGF and promote the wound healing of mice with deep second-degree scald. Adv. Healthc. Mater. 6, 1700344. 10.1002/adhm.201700344 28661050

[B162] Xu Z. Shi L. Yang M. Zhu L. (2019). Preparation and biomedical applications of silk fibroin-nanoparticles composites with enhanced properties - a review. Mater. Sci. Eng. C, Mater. Biol. Appl. 95, 302–311. 10.1016/j.msec.2018.11.010 30573254

[B163] Yan L. P. Silva-Correia J. Ribeiro V. P. Miranda-Gonçalves V. Correia C. da Silva Morais A. (2016). Tumor growth suppression induced by biomimetic silk fibroin hydrogels. Sci. Rep. 6, 31037. 10.1038/srep31037 27485515 PMC4971568

[B164] Yang R. Hou M. Gao Y. Lu S. Zhang L. Xu Z. (2019). Biomineralization-inspired crystallization of manganese oxide on silk fibroin nanoparticles for *in vivo* MR/fluorescence imaging-assisted Tri-modal therapy of cancer. Theranostics 9, 6314–6333. 10.7150/thno.36252 31534553 PMC6735506

[B165] Yao Q. Lan Q. H. Jiang X. Du C. C. Zhai Y. Y. Shen X. (2020). Bioinspired biliverdin/silk fibroin hydrogel for antiglioma photothermal therapy and wound healing. Theranostics 10, 11719–11736. 10.7150/thno.47682 33052243 PMC7545989

[B166] Yucel T. Lovett M. L. Giangregorio R. Coonahan E. Kaplan D. L. (2014). Silk fibroin rods for sustained delivery of breast cancer therapeutics. Biomaterials 35, 8613–8620. 10.1016/j.biomaterials.2014.06.030 25009069

[B167] Zha X. Xiong X. Chen C. Li Y. Zhang L. Xie H. (2021). Usnic-acid-functionalized silk fibroin composite scaffolds for cutaneous wounds healing. Macromol. Biosci. 21, e2000361. 10.1002/mabi.202000361 33369081

[B168] Zhang D. Chu Y. Qian H. Qian L. Shao J. Xu Q. (2020a). Antitumor activity of thermosensitive hydrogels packaging gambogic acid nanoparticles and tumor-penetrating peptide iRGD against gastric cancer. Int. J. Nanomedicine 15, 735–747. 10.2147/IJN.S231448 32099362 PMC6999774

[B169] Zhang W. Zheng Y. Liu H. Zhu X. Gu Y. Lan Y. (2019). A non-invasive monitoring of USPIO labeled silk fibroin/hydroxyapatite scaffold loaded DPSCs for dental pulp regeneration. Mater. Sci. Eng. C, Mater. Biol. Appl. 103, 109736. 10.1016/j.msec.2019.05.021 31349524

[B170] Zhang Y.-Q. Shen W.-D. Xiang R.-L. Zhuge L.-J. Gao W.-J. Wang W.-B. (2007). Formation of silk fibroin nanoparticles in water-miscible organic solvent and their characterization. J. Nanoparticle Res. 9, 885–900. 10.1007/s11051-006-9162-x

[B171] Zhang Y. Cao Y. Zhang L. Zhao H. Ni T. Liu Y. (2020b). Fabrication of an injectable BMSC-laden double network hydrogel based on silk fibroin/PEG for cartilage repair. J. Mater. Chem. B 8, 5845–5848. 10.1039/d0tb01017k 32667029

[B172] Zhao M. Zhu T. Chen J. Cui Y. Zhang X. Lee R. J. (2021). PLGA/PCADK composite microspheres containing hyaluronic acid-chitosan siRNA nanoparticles: A rational design for rheumatoid arthritis therapy. Int. J. Pharm. 596, 120204. 10.1016/j.ijpharm.2021.120204 33493604

[B173] Zhao Y. Z. Lin M. T. Lan Q. H. Zhai Y. Y. Xu H. L. Xiao J. (2020). Silk fibroin-modified disulfiram/zinc oxide nanocomposites for pH triggered release of Zn(2+) and synergistic antitumor efficacy. Mol. Pharm. 17, 3857–3869. 10.1021/acs.molpharmaceut.0c00604 32833457

[B174] Zhao Z. Chen A. Li Y. Hu J. Liu X. Li J. (2012). Fabrication of silk fibroin nanoparticles for controlled drug delivery. J. Nanoparticle Res. 14, 736. 10.1007/s11051-012-0736-5

[B175] Zhao Z. Li Y. Xie M. B. (2015). Silk fibroin-based nanoparticles for drug delivery. Int. J. Mol. Sci. 16, 4880–4903. 10.3390/ijms16034880 25749470 PMC4394455

[B176] Zheng H. Zuo B. (2021). Functional silk fibroin hydrogels: Preparation, properties and applications. J. Mater. Chem. B 9, 1238–1258. 10.1039/d0tb02099k 33406183

[B177] Zheng Z. Guo S. Liu Y. Wu J. Li G. Liu M. (2016). Lithium-free processing of silk fibroin. J. biomaterials Appl. 31, 450–463. 10.1177/0885328216653259 27298185

[B178] Zhu M. Liu Y. Jiang F. Cao J. Kundu S. C. Lu S. (2020). Combined silk fibroin microneedles for insulin delivery. ACS Biomaterials Sci. Eng. 6, 3422–3429. 10.1021/acsbiomaterials.0c00273 33463180

[B179] ZhuGe D. L. Wang L. F. Chen R. Li X. Z. Huang Z. W. Yao Q. (2019). Cross-linked nanoparticles of silk fibroin with proanthocyanidins as a promising vehicle of indocyanine green for photo-thermal therapy of glioma. Artif. Cells Nanomed Biotechnol. 47, 4293–4304. 10.1080/21691401.2019.1699819 31810396

[B180] Ziadlou R. Rotman S. Teuschl A. Salzer E. Barbero A. Martin I. (2021). Optimization of hyaluronic acid-tyramine/silk-fibroin composite hydrogels for cartilage tissue engineering and delivery of anti-inflammatory and anabolic drugs. Mater. Sci. Eng. C, Mater. Biol. Appl. 120, 111701. 10.1016/j.msec.2020.111701 33545860

[B181] Zou X. Jiang Z. Li L. Huang Z. (2021). Selenium nanoparticles coated with pH responsive silk fibroin complex for fingolimod release and enhanced targeting in thyroid cancer. Artif. Cells Nanomed Biotechnol. 49, 83–95. 10.1080/21691401.2021.1871620 33438446

[B182] Zuluaga-Vélez A. Quintero-Martinez A. Orozco L. M. Sepúlveda-Arias J. C. (2021). Silk fibroin nanocomposites as tissue engineering scaffolds - a systematic review. Biomed. Pharmacother. 141, 111924. 10.1016/j.biopha.2021.111924 34328093

